# Shining a Spotlight on Methyl Groups: Photochemically Induced Dynamic Nuclear Polarization Spectroscopy of 5-Deazariboflavin and Its Nor Analogs

**DOI:** 10.3390/ijms25020848

**Published:** 2024-01-10

**Authors:** Sabrina Panter, Audrey Ayekoi, Jannis Tesche, Jing Chen, Boris Illarionov, Adelbert Bacher, Markus Fischer, Stefan Weber

**Affiliations:** 1 Institut für Physikalische Chemie, Albert-Ludwigs-Universität Freiburg, Albertstr. 21, 79104 Freiburg, Germany; sabrina.panter@pc.uni-freiburg.de (S.P.); audrey.ayekoi@pc.uni-freiburg.de (A.A.);; 2 Institut für Lebensmittelchemie, Universität Hamburg, Grindelallee 117, 20146 Hamburg, Germany; boris.illarionov@uni-hamburg.de (B.I.); markus.fischer@uni-hamburg.de (M.F.); 3 TUM School of Natural Sciences, Technische Universität München, Lichtenbergstr. 4, 85747 Garching, Germany; adelbert.bacher@t-online.de

**Keywords:** 5-deazaflavin, photo-CIDNP, hyperpolarization, spin-correlated radical pair, hyperfine coupling

## Abstract

5-Deazaflavins are analogs of naturally occurring flavin cofactors. They serve as substitutes for natural flavin cofactors to investigate and modify the reaction pathways of flavoproteins. Demethylated 5-deazaflavins are potential candidates for artificial cofactors, allowing us to fine-tune the reaction kinetics and absorption characteristics of flavoproteins. In this contribution, demethylated 5-deazariboflavin radicals are investigated (1) to assess the influence of the methyl groups on the electronic structure of the 5-deazaflavin radical and (2) to explore their photophysical properties with regard to their potential as artificial cofactors. We determined the proton hyperfine structure of demethylated 5-deazariboflavins using photochemically induced dynamic nuclear polarization (photo-CIDNP) spectroscopy, as well as density functional theory (DFT). To provide context, we compare our findings to a study of flavin mononucleotide (FMN) derivatives. We found a significant influence of the methylation pattern on the absorption properties, as well as on the proton hyperfine coupling ratios of the xylene moiety, which appears to be solvent-dependent. This effect is enhanced by the replacement of N5 by C5-H in 5-deazaflavin derivatives compared to their respective flavin counterparts.

## 1. Introduction

**Figure 1:**
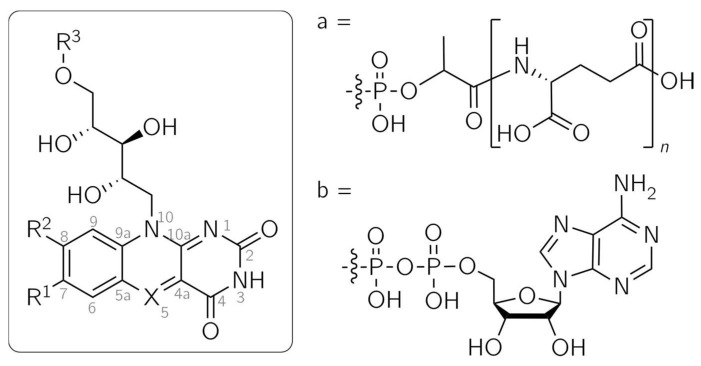
Structures of flavin and 5-deazaflavin analogs. F_o_: X = CH, R^1^ = R^3^ = H, R^2^ = OH; F_420_: X = CH, R^1^ = H, R^2^ = OH, R^3^ = a; Flavin mononucleotide (FMN): X = N, R^1^ = R^2^ = Me, R^3^ = PO_3_H_2_; 5-DeazaFMN: X = CH, R^1^ = R^2^ = Me, R^3^ = PO_3_H_2_; 5-Deazaflavin adenine dinucleotide (5-DeazaFAD): X = CH, R^1^ = R^2^ = Me, R^3^ = b; 5-Deazariboflavin: X = CH, R^1^ = R^2^ = Me, R^3^ = H.

5-Deazaflavins are analogs of the widely spread flavins, which serve as protein cofactors in a vast number of biochemical processes. While not as abundantly distributed, 5-deazaflavins can be found as the natural compounds F_420_ and F_o_, the latter being also known as 7,8-didemethyl-8-hydroxy-5-deazariboflavin [1,2,3,4] (Figure 1). The occurrence of F_420_ was first reported in 1972 as a coenzyme in the *Methanobacterium* strain *Methanobacterium omelianskii* (M.o.H.) [5,6] and has since been detected in Gram-positive eubacteria and archaea [3], where they catalyze a range of redox reactions. They are involved in processes such as methanogenesis [7]. F_o_ functions as a second, light-harvesting chromophore. It is among others found in the cryptochrome *Cr*aCRY from the green alga *Chlamydomonas reinhardtii* [8] and in DNA photolyase, a DNA-repair enzyme [9,10], where it serves to improve the blue-light sensitivity of the protein.

Flavins and 5-deazaflavins share similar structural features, as only position 5 is altered from N in flavins to C-H in 5-deazaflavins (Figure 1). This modification yields substantially differing photochemical and electrochemical reactivities, opening up possible applications: due to the stability of the reduced 5-deazaflavins toward oxygen, they are promising alternatives to flavins as redox-active photocatalysts for the direct, nicotinamide-independent regeneration of flavin-based monooxygenases [11]. Moreover, 5-deazaflavins are excellent replacement cofactors for investigating the reaction mechanisms of flavoproteins [12]. Experiments with different flavoproteins led to the observation that single-electron transfer processes are usually prevented by the replacement of the flavin cofactor with the 5-deazaflavin analog. Different functions of flavoproteins, such as oxygen activation and electron transfer, are frequently impeded, leaving only (de)hydrogenation as the sole functioning catalytic role [12]. 

It has been demonstrated that even the functionality of a flavoprotein can be substantially altered through cofactor replacement with a 5-deazaflavin analog. The recently published transformation of a dehalogenase into a nitrogenase [13] serves as an astonishing example. In the blue-light receptor domain LOV2 (light, oxygen, voltage) of phot1, a photo-switch protein was generated by inserting a 5-deazaflavin mononucleotide (5-deazaFMN) cofactor (Figure 1). These studies were conducted in our laboratories [14] and applied to a similar protein, the YtvA protein from *Bacillus subtilis*, by Silva-Junior et al. [15]. The wild-type LOV protein undergoes a reaction cycle initiated by light irradiation: after the excitation of the flavin cofactor into a triplet state via intersystem crossing from an excited singlet state, a photoadduct between C4a of the flavin and a close cysteine residue of the protein chain is formed [15,16]. The recovery of the dark state is thermally driven. In the LOV proteins phot1 and phot2 from *Arabidopsis thaliana*, the photoadduct’s lifetime covers several seconds, while it is extended to 10,000 s in *Neurospora crassa* VIVID [17,18,19]. By exchanging the cofactor with 5-deazaFMN, the lifetime is prolonged to several days, and the recovery of the dark state is achieved by photoexciting the adduct [14,15]. The adduct formation and dark-state recovery of 5-deazaFMN in the YtvA protein were further analyzed using optical spectroscopy and quantum mechanics/molecular mechanics (QM/MM) calculations. After light excitation into the S_1_ state and subsequent intersystem crossing into the T_1_ state, hydrogen transfer from the thiol group to C5 of 5-deazaFMN occurs. The biradical undergoes intersystem crossing into the S_0_ ground state, and adduct formation via a C4a-S bond takes place. The photoinduced-dark-state recovery is predicted to be only accessible through a C4a-adduct [15]. This photo-switching between dark and adduct states can be repeated several times without a significant loss of efficiency [14,15].

Due to the observation that single-electron transfer reactions in flavoproteins are frequently impeded by the non-native 5-deazaflavin cofactor, the 5-deazaflavin radical was long believed to be too unstable to be detected [20,21]. A continuous-wave (cw) electron paramagnetic resonance (EPR) spectrum assigned to a 5-deazaflavin adenine dinucleotide (5-deazaFAD) radical was reported in 1977, albeit with a poor signal-to-noise-ratio [22]. Since no spectral simulation was attempted and there is a lack of additional data from other EPR techniques, this cannot be considered conclusive evidence of the 5-deazaflavin radical. Recently, a radical based on a C5-phenylated 5-deazaflavin derivative, a promising reductive photocatalyst, was detected by cw EPR. The compound has further modifications at positions 7 and 8 (OCH_3_ instead of CH_3_), 3 (CH_3_ instead of H) and 10 (CH_3_ instead of ribityl). The spectral simulation yielded three substantial hyperfine couplings that were assigned to N10 and protons 2′ and 6′ of the phenyl substituent [23]. In a study conducted in our laboratories [24], we demonstrated that the 5-deazaflavin mononucleotide (5-deazaFMN) radical is indeed detectable under physiological conditions. 

The detection of the 5-deazaFMN radical was accomplished using photochemically induced dynamic nuclear polarization (photo-CIDNP) spectroscopy. This NMR technique can be used to probe short-lived radical pairs [25,26,27] that are difficult to detect by EPR techniques [27,28,29,30]. The electron spin density is transferred to the nuclei of the light-induced radical pair in a spin-sorting process, which results in enhanced and diminished NMR resonances [31,32,33]. This process is based on the multiplicity-dependent reaction pathway and on the nuclear-spin-dependent singlet–triplet mixing of the spin-correlated radical pair. With the time-resolved version of photo-CIDNP spectroscopy, the determination of isotropic hyperfine coupling constants is possible, and information on the mechanistic details of radical-pair formation is accessible [34]. This enables a highly sensitive mapping of the hyperfine structure of the probed spin-correlated radical pair, even for weak hyperfine couplings [28,34,35,36]. 

The photo-CIDNP effect is explained by the radical-pair mechanism [31,37]: the acceptor moiety, e.g., a flavin, is photoexcited into an excited singlet or triplet state. Electron transfer from a donor molecule, e.g., an aromatic amino acid, yields a spin-correlated radical pair whose multiplicity corresponds to the precursor’s multiplicity. The radical pair proceeds to oscillate between the triplet and singlet states. The frequency of this oscillation depends, among other factors, on the difference in isotropic *g* factors of the radical pair and their hyperfine couplings. Simultaneously, in an isotropic environment, the radical pair diffuses apart. The recombination of a singlet radical pair can take place after reencountering to form a so-called “recombination product”, while triplet radical pairs tend to separate and form “escape products”. These parallel processes result in spin sorting, as certain nuclear spin configurations are favored by different reaction routes and thus are enriched in the recombination or escape product [26,27,34,38]. A simple relationship, called “Kaptein’s rule”, enables the determination of the sign of the resulting CIDNP signal [39]:

*Γ_i_* = *μ* × *ε* × sgn(Δ*g*) × sgn(*A*_iso,*i*_). (1)

The sign *Γ_i_* can either be absorptive (“+”) or emissive (“−”). *μ* denotes the multiplicity of the precursor, which is either triplet (“+”) or singlet (“−”). The parameter *ε* is determined by the route of product formation. The formation of a recombination product yields “+”, while the observation of an escape product gives “−”. Additionally, the sign of the difference between the *g* factors, Δ*g* = *g*_iso,1_ − *g*_iso,2_, of the radicals forming the radical pair, as well as the sign of the isotropic hyperfine coupling constant *A*_iso,*i*_, have to be taken into account.

After the recent detection of the 5-deazaFMN radical and the investigation of its proton hyperfine interactions [24], we wanted to further examine its hyperfine structure and assess the influence of the methyl groups at positions 7 and 8. In particular, the 7α methyl group in the FMN semiquinone shows a significant impact on the ratio between the hyperfine couplings at positions 6 and 7 [29].

In order to describe and understand protein–cofactor interactions at specific parts of the cofactor, mutations in the amino acid chain close to the site of the flavin cofactor can be inserted [40,41]. A complementary approach is the incorporation of modified cofactors, for which demethylated 5-deazariboflavins (Figure 1) are potential alternatives. Furthermore, the use of this group of 5-deazariboflavins makes it possible to alter reaction kinetics and tune absorption properties to the desired values, as has been demonstrated by Mansurova et al. with flavin derivatives [42]. For this purpose, a thorough understanding of their electronic structures is invaluable.

In this work, 5-deazariboflavin and its demethylated analogs were studied using optical and photo-CIDNP spectroscopy to assess the influence of the methylation pattern on the absorption properties of 5-deazariboflavin and on the electronic structure of the respective radical. The experiments were conducted in both DMSO and aqueous solution to compare the radicals in different solvent environments. By using density functional theory (DFT), the values of the hyperfine coupling and *g* factors were calculated and compared to the experimental data.

## 2. Results

### 2.1. Absorption Properties

**Figure 2:**
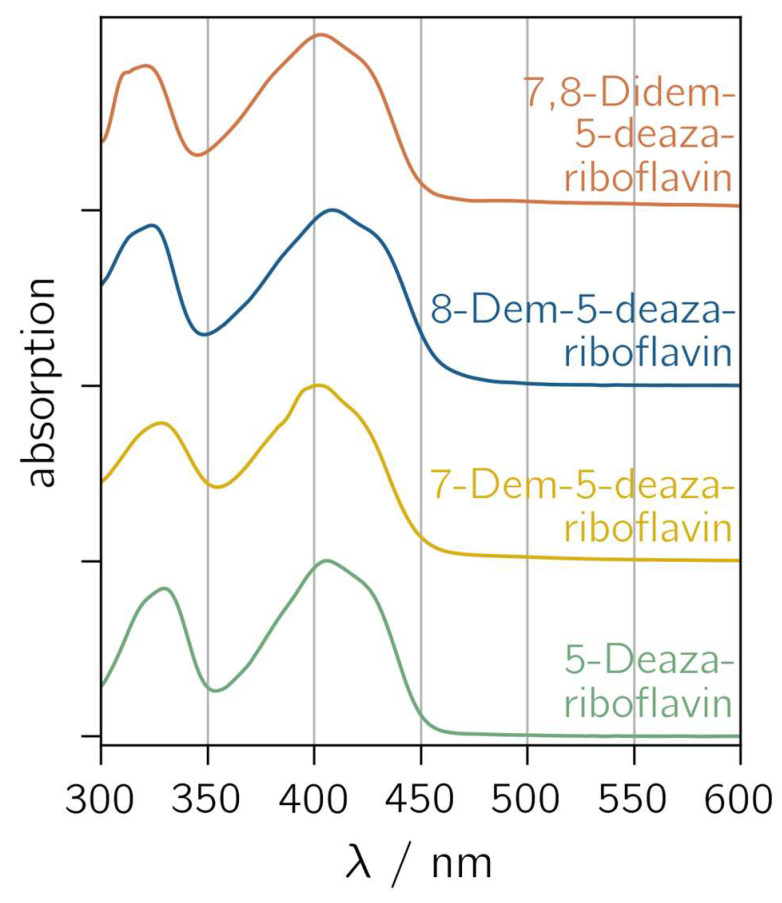
Normalized absorption spectra of 5-deazariboflavin and demethylated derivatives in DMSO. “Demethyl” is abbreviated “dem”.

5-Deazariboflavin and its nor derivatives were synthesized according to the scheme outlined in Figure S1. The absorption spectra of 5-deazariboflavin and the nor analogs 7-demethyl-5-deazariboflavin, 8-demethyl-5-deazariboflavin and 7,8-didemethyl-5-deazariboflavin are shown in Figure 2. All derivatives exhibit two characteristic absorption bands with maxima around 400 nm and 330 nm. Both absorption bands can be assigned to π→π* transitions according to time-dependent DFT (TD-DFT) calculations [43]. Compared to riboflavin, the absorption maxima of 5-deazaflavins are shifted hypsochromically [44] (407 nm for 5-deazariboflavin compared to 448 nm for riboflavin [45]). This shift can be mainly attributed to a destabilization of the lowest occupied molecular orbital (LUMO) due to the exchange of N with C-H at position 5 [43].

The removal of the 8-methyl group induces a hypsochromic shift in the short-wavelength absorption maximum (323 nm and 321 nm for 8-demethyl-5-deazariboflavin and 7,8-didemethyl-5-deazariboflavin, respectively, compared to 330 nm for 5-deazariboflavin), whereas the lack of the 7-methyl group does not affect the absorption band maximum significantly (328 nm for 7-demethyl-5-deazariboflavin). The position of the long-wavelength absorption maximum exhibits an inverse dependency: after the removal of the 7-methyl group, a hypsochromic shift is observed (402 nm and 403 nm for 7-demethyl-5-deazariboflavin and 7,8-didemethyl-5-deazariboflavin, respectively, compared to 407 nm for 5-deazariboflavin), while for 8-methyl-5-deazariboflavin, an absorption maximum of 408 nm is measured.

A comparison of the absorption properties in DMSO with those in aqueous solution (Figure S2 and Table S1) shows a weak solvent dependency for all 5-deazariboflavin analogs. The long-wavelength absorption maximum is blue-shifted by 6–12 nm in aqueous solution, while the short-wavelength absorption maximum is red-shifted by 6–12 nm, resulting in the diminished distance of both absorption bands compared to the absorption spectra in Figure 2.

### 2.2. Investigation of Demethylated 5-Deazariboflavin Radicals

#### 2.2.1. Transient Photo-CIDNP Experiments of Demethylated 5-Deazariboflavin Derivatives

**Figure 3:**
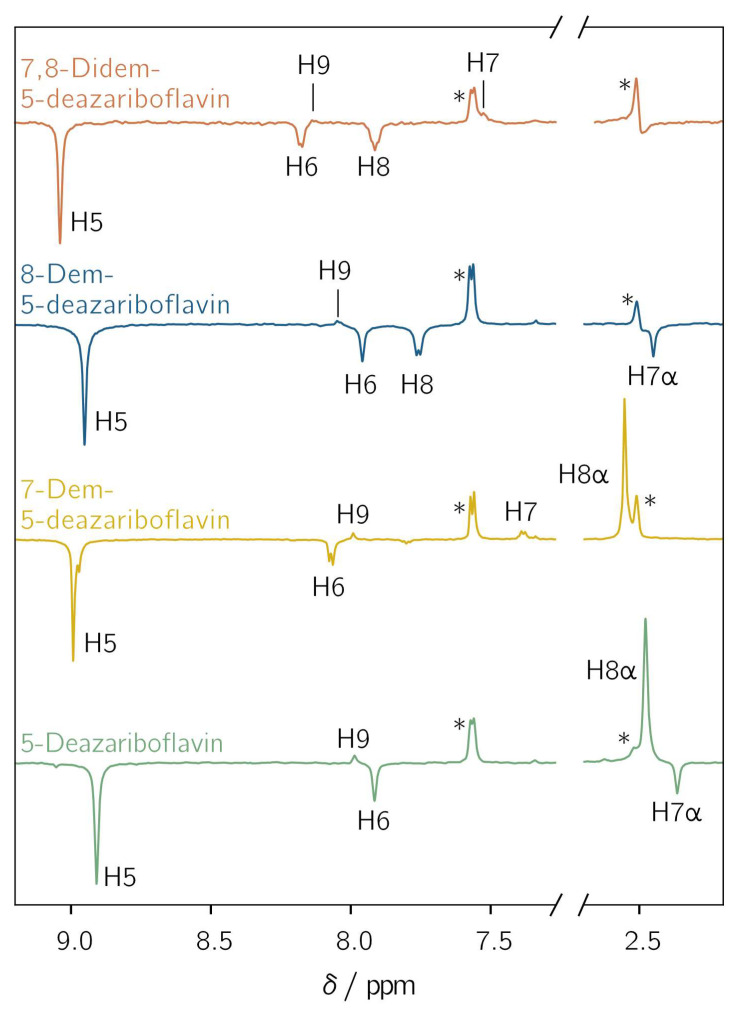
Transient ^1^H photochemically induced dynamic nuclear polarization (photo-CIDNP) spectra of 5-deazariboflavin (green), 7-demethyl-5-deazariboflavin (yellow), 8-demethyl-5-deazariboflavin (blue) and 7,8-didemethyl-5-deazariboflavin (orange). The concentrations of the 5-deazariboflavin derivative and l-tryptophan were 0.25 mm and 5 mM, respectively. The samples were irradiated at 420 nm with laser pulse energies of 4.6 to 6.8 mJ. The ^1^H resonances of the ribityl chain are not shown. Asterisks denote the resonances of H4 of l-tryptophan at 7.6 ppm and the residual DMSO-d_6_ signal at 2.5 ppm. All spectra were referenced to the latter resonance.

All demethylated 5-deazariboflavin analogs were investigated with l-tryptophan as an electron donor in DMSO-d_6_ using photo-CIDNP NMR with pulsed photoexcitation. Hyperpolarized proton NMR resonances assigned to the protons of the 5-deazaisoalloxazine moiety are observed for all derivatives (Figure 3). The observation of hyperpolarized resonances provides clear and unambiguous evidence of the generation of a spin-correlated radical pair after photoinduced electron or hydrogen transfer. Positive and negative resonances correspond to absorptive and emissive transitions, respectively. The protons exhibit a similar hyperpolarization pattern regardless of the investigated derivative. For H5, H6, H7α and H8, only emissive resonances are observed, whereas for H7, H8α, and H9, absorptive resonances are found. For positions 7 and 8, a sign change is observed upon demethylation: the 7α-methyl group shows an emissive CIDNP resonance, while H7 is absorptive. For position 8, the CIDNP sign phase changes from absorptive for the 8α-methyl group to emissive for H8 after demethylation. This is due to different mechanisms for the spin-density transfer. For an aromatic proton, the spin density is transferred directly from the adjacent aromatic carbon through exchange coupling between *π* and *σ* electrons [46], while hyperconjugation is responsible for the spin-density transfer to α protons [47].

Two further protons are (potentially) present in the 5-deazaisoalloxazine moiety: (i) A proton is bound to N1 if radical-pair formation proceeds via hydrogen transfer from the tryptophanyl donor. The protonation of position 5, as it is the case for flavins, is not possible due to the formation of an unstable radical species. Thus, only N1 is available as a protonation site [48]. A CIDNP signal arising from this proton is not observed because its hyperfine coupling is expected to be close to zero (see Table S2). Furthermore, radical-pair formation involving tryptophan usually proceeds via electron transfer, resulting in an anionic flavin radical species [49]. (ii) For H3, a weak hyperfine coupling is predicted for each considered derivative (Table S2). This proton does not show any hyperpolarized resonance. Thus, the nucleus is not further considered.

#### 2.2.2. Mechanism of the Radical-Pair Formation of Demethylated 5-Deazariboflavin Derivatives

The electron-spin multiplicity of the radical pair’s precursor can be determined using Kaptein’s rule (see Equation (1)). If the radical pair is formed upon electron transfer from l-tryptophan, the positive tryptophanyl radical TrpH^●+^ and the negative 5-deazariboflavin radical 5-deazariboflavin^●−^ are formed. Upon hydrogen transfer, neutral radical species, Trp^●^ and 5-deazariboflavin(H1)^●^, are to be expected, the latter being protonated at N1 [24]. l-Tryptophan is known to be an electron donor in photoinduced electron transfer reactions [49], which is why the formation of a [TrpH^●+^ 5-Deazariboflavin^●−^] radical pair is probable. The subsequent protonation of 5-deazariboflavin^●−^ is not possible, as no acidic protons are available in the DMSO environment.

Experimental data on the isotropic *g* factors of demethylated 5-deazariboflavin radicals are not available. Therefore, DFT calculations were employed (Table 1), giving *g* factors ranging from 2.00274 to 2.00283 for 5-deazariboflavin radical derivatives. The *g* factor of Trp^●^ was determined experimentally to be 2.0026 [50], while there are no experimental data available for the *g* factor of TrpH^●+^. DFT-based studies give a value of 2.00280 [51]. The latter is in the range of the calculated *g* factors of 5-deazariboflavin derivatives. An exact determination of sgn(Δ*g*) is therefore not possible based on these data, as subtle changes in the structure used for the DFT calculations could result in deviations for the predicted *g* factors and a change in sgn(Δ*g*). For this reason, additional experiments were conducted in D_2_O with l-tyrosine as an electron donor, although poorer solubility limits the detection of CIDNP signals (see Figures S3–S5).

To determine the radical pair precursor’s multiplicity *μ*, the parameters of Equation (1) regarding the photo-CIDNP experiments in D_2_O are required. They were determined as follows. (i) l-Tyrosine radicals in both relevant protonation states yield *g* factors of ~2.0045 [52,53] that are significantly larger than the values calculated for the demethylated 5-deazariboflavin radicals (see Table S3 for results of calculations using implicit water solvation). Thus, sgn(Δ*g*) for the radical pair can be determined unequivocally to be “−“. (ii) Isotropic hyperfine coupling constants *A*_iso_ are taken from DFT calculations as well (see Table S4). For the protons H5, H6, H7α and H8, sgn(*A*_iso,*i*_) = “−”, whereas sgn(*A*_iso_) = “+” for H7, H8α, and H9 for all 5-deazariboflavin derivatives. (iii) ^1^H photo-CIDNP resonance phases of 5-deazariboflavin derivatives are inverted for experiments with l-tyrosine as the electron donor compared to experiments with l-tryptophan as the electron donor (compare Figure 3 and Figures S3–S5). Absorptive resonances are obtained for H5, H6, H7α and H8 (i.e., *Γ_i_* = “+”), and emissive resonances are obtained for H7, H8α and H9 (i.e., *Γ_i_* = “−”). Comparing the calculated *A*_iso_ and CIDNP signal phases of all derivatives indicates that *Γ_i_* and *A*_iso,*i*_ have opposite signs. (iv) Using the transient photo-CIDNP variant, the time resolution of the experiment is only determined by the length of the radio frequency probe pulse of 2.5 μs. In this time frame, the processes responsible for secondary CIDNP kinetics are not active [34]. Thus, the CIDNP resonances can only arise from geminate CIDNP polarization, resulting in *ε* = “+”. (v) The above considerations yield *Γ_i_* = *μ* × “+” × “−” × −sgn(*A*_iso,*i*_), which requires *μ* = “+”; thus, the radical pair of all investigated 5-deazariboflavin derivatives is formed after the photoexcitation of 5-deazariboflavin into a triplet state via intersystem crossing.

#### 2.2.3. Determination of Isotropic Proton Hyperfine Coupling Constants

For the determination of relative isotropic hyperfine couplings, proton CIDNP intensities were extracted by fitting a Voigt profile, if not indicated otherwise, to the CIDNP signals of the demethylated 5-deazariboflavin analogs in Figure 3. A proper fit was not possible for H7 of 7,8-didemethyl-5-deazariboflavin due to the poor signal-to-noise ratio. For 7-demethyl-5-deazariboflavin, the fitting of the hyperpolarized resonance of H5 could be performed well, despite the partial superposition of a second hyperpolarized resonance from a different, unidentified flavin species. Table 2 lists the experimental results, as well as the absolute and relative hyperfine couplings of the relevant ^1^H nuclei from DFT calculations.

The relative CIDNP intensities and the calculated hyperfine couplings deviate only slightly. Specifically, the hyperfine coupling of H8 of the 8-demethyl-5-deazariboflavin radical is stronger than that calculated by DFT. The hyperfine coupling of H9 is predicted to be slightly too high for the radical species of 5-deazariboflavin, 8-demethyl-5-deazariboflavin and 7,8-demethyl-5-deazariboflavin. For H7α of 8-demethyl-5-deazariboflavin, DFT predicted a stronger hyperfine coupling, as well.

**Figure 4:**
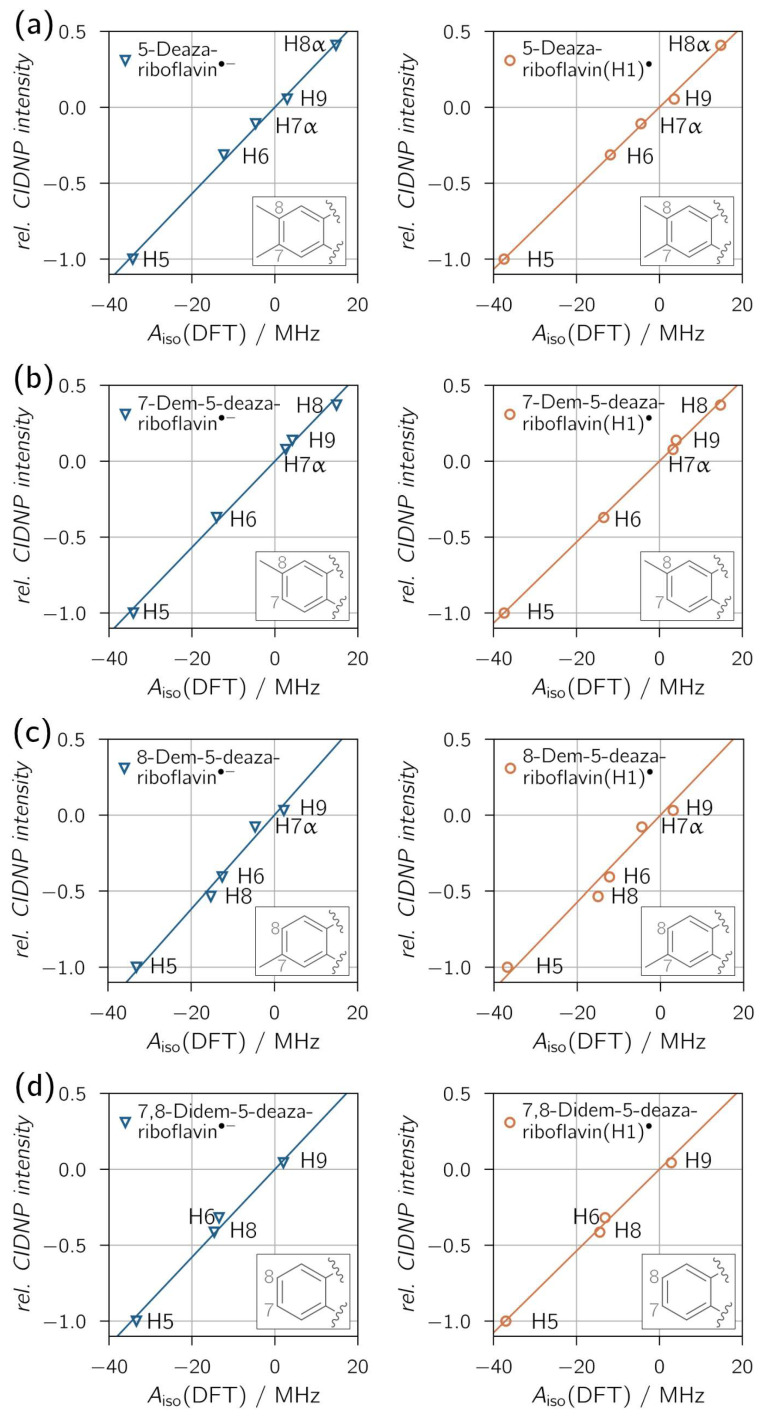
Correlation plots of ^1^H transient CIDNP experimental results of (**a**) 5-deazariboflavin, (**b**) 7-demethyl-5-deazariboflavin, (**c**) 8-demethyl-5-deazariboflavin and (**d**) 7,8-didemethyl-5-deazariboflavin in DMSO-d_6_. Linear fits were forced to go through the origin. The following values for the slope *m* and *R*^2^ were found for the respective anionic (left column) and neutral (right column) radical species, respectively: (**a**) *m* = 0.0285 MHz^−1^, *R*^2^ = 0.9967; *m* = 0.0267 MHz^−1^, *R*^2^ = 0.9982; (**b**) *m* = 0.0284 MHz^−1^, *R*^2^ = 0.9959; *m* = 0.0267 MHz^−1^, *R*^2^ = 0.9986; (**c**) *m* = 0.0309 MHz^−1^, *R*^2^ = 0.9839; *m* = 0.0286 MHz^−1^, *R*^2^ = 0.9657; (**d**) *m* = 0.0289 MHz^−1^, *R*^2^ = 0.9885; *m* = 0.0269 MHz^−1^, *R*^2^ = 0.9946.

Figure 4 shows the correlations between relative proton CIDNP intensities and DFT-predicted hyperfine couplings, with overall exceptional fits reflected by high *R*^2^ values. A clear distinction between neutral (protonated) and anionic radical species is not possible, as all derivatives show similar proton hyperfine coupling patterns for both protonation states. Only for 8-demethyl-5-deazariboflavin the *R*^2^ values are distinct enough, with *R*^2^ = 0.9657 for 8-demethyl-5-deazariboflavin(H1)^●^ and *R*^2^ = 0.9839 for 8-demethyl-5-deazariboflavin^●−^, to provide evidence for radical-pair formation via electron transfer.

Additional experiments in D_2_O were performed at pH values of about 1.7 and 8.0 (see Figures S4 and S5 for thermal and CIDNP spectra). Relative CIDNP intensities extracted from these experiments, as well as calculated hyperfine coupling constants using implicit water solvation, are listed in Table S4. For the 5-deazalumiflavin triplet state and the 5-deazalumiflavin radical, the p*K*_A_ values were determined to be <3 [54] and 6 [55], respectively. Adjusting the pH value of the samples to approximately 1.7 and 8.0 should allow the measurement of CIDNP polarization from one specific protonation state. The correlations between relative CIDNP intensities and hyperfine coupling constants (see Figures S6–S9) are close to 1. The similarity of *R*^2^ for experiments with different pH does not permit a clear distinction between the protonation states for any of the demethylated 5-deazariboflavin derivatives.

Positions 7 and 8 seem to have a particular influence on the proton hyperfine pattern of the 5-deazaisoalloxazine moiety, more specifically on the ratios *A*_iso,H6_:*A*_iso,H8_ and *A*_iso,H9_:*A*_iso,H8_, respectively. A seemingly solvent-dependent effect complicates a straightforward analysis (compare CIDNP intensities in Table 2 and Table S4). 7-Demethyl-5-deazariboflavin solvated in DMSO-d_6_ shows an elevated hyperfine coupling ratio *A*_iso,H6_:*A*_iso,H8α_, as well as *A*_iso,H6_:*A*_iso,H9_, compared to the other investigated derivatives. In D_2_O, similar deviations are obtained for 7-demethyl-5-deazariboflavin at both pH values. 8-Demethyl-5-deazariboflavin shows the same behavior at pH 1.5, while at pH 7.6, only *A*_iso,H6_:*A*_iso,H8_ is elevated. A direct comparison with 7,8-didemethyl-5-deazariboflavin is difficult, as the hyperfine coupling constants of H6 and H9 could not be determined in aqueous solution due to signal overlap. Nevertheless, both methyl groups show a significant effect on the proton hyperfine structure and, consequently, on the spin-density map of the 5-deazaisoalloxazine structure, mainly at positions 6 and 9.

### 2.3. Spin-Density Distributions of Demethylated 5-Deazariboflavin and Riboflavin Radicals

According to McConnell and Chesnut [46], aromatic proton hyperfine couplings in π-system radicals depend on the spin density of the respective adjacent aromatic carbon. For α protons, a sign change is expected, while the size of hyperfine coupling is comparable to the hyperfine coupling expected for a proton adjacent to the aromatic carbon [47]. This relation can be confirmed by comparing the experimental relative hyperfine couplings and the calculated spin densities for all 5-deazariboflavin derivatives (see Table S5 for the calculation results). The predicted spin densities for C6, C7, C8 and C9 show a negligible dependence on the methylation pattern of 5-deazariboflavin and do not reflect the effect of demethylation on the proton hyperfine pattern found in 7-demethyl-5-deazariboflavin and 8-demethyl-5-deazariboflavin discussed above.

The spin densities of demethylated riboflavin radical derivatives were calculated, as well (Table S6). A comparison of 5-deazariboflavin and riboflavin derivatives reveals the effects induced by the atom at position 5: C-H or N. The spin-density values in Tables S5 and S6 show that the relative spin-density patterns and the signs thereof remain unaffected by the exchange of N5 with C5-H. However, in 5-deazariboflavin radical derivatives, nuclei with a negative spin density exhibit more negative values, while nuclei with a positive spin density show more positive values. Due to the protonation of N5, the spin-density distribution of riboflavin derivatives is altered substantially, resulting in less negative and less positive spin densities in the carbon and nitrogen framework, with the exception of C4a and N10. As the protonation site N1 is farther away from the xylene moiety, the protonation of 5-deazariboflavin derivatives only affects closer atoms, in particular, C10a, C4a, C4 and C5, but leaves atoms of the xylene moiety mostly unaffected. This results in only small deviations of proton hyperfine couplings upon protonation. Thus, for both protonation states, 5-deazariboflavin radical derivatives share a similar spin-density distribution with the anionic riboflavin radical derivatives. For H9, almost identical spin densities are found. On the contrary, the protonated riboflavin radical derivatives are more distinct from the respective anionic radical. These findings agree with the results of our photo-CIDNP study. Pompe et al. [29] were able to distinguish between anionic and cationic radical species of FMN derivatives. As shown above, the same is not possible for 5-deazariboflavin derivatives using ^1^H photo-CIDNP spectroscopy, as the hyperfine patterns of anionic and cationic radical species do not differ significantly.

## 3. Discussion

The determination of absorption properties shows a clear dependence on the methylation pattern. Similar effects were reported for alloxazines and isoalloxazines in acetonitrile [56] and for demethylated FMN analogs in aqueous solutions [29]. For demethylated FMN analogs, shifts in the absorption maxima were found that were similar to the ones described in this contribution: The long-wavelength absorption maximum solely depends on the presence of the 7-methyl group. The short-wavelength absorption maximum, although it is a superposition of transitions with σ→π*, n→π* and π→π* character [57], as opposed to a π→π* transition for 5-deazariboflavin derivatives [43], is only determined by the 8-methyl group. The reported shifts in the absorption bands after demethylation are comparable in size to those described for demethylated 5-deazariboflavin derivatives, indicating that exchanging N5 with C5-H does not induce an additional shift in the absorption bands.

The investigated 5-deazariboflavin derivatives show a significant CIDNP effect while retaining similar photophysics compared to 5-deazaFMN [24]: the discussed CIDNP spectra with l-tyrosine as the electron donor provide clear evidence for radical-pair formation via an excited 5-deazaflavin triplet. This places them as potential alternatives to flavins and 5-deazaflavins in cofactor replacement experiments with flavoproteins. As all derivatives are able to undergo radical reactions, their application is expanded to flavoproteins catalyzing radical reactions. 7,8-Didemethyl-5-deazariboflavin could be of extended use for replacement experiments that are investigated using NMR spectroscopy, as this derivative offers cheaper options for the ^13^C labeling of the 5-deazaisoalloxazine moiety compared to 5-deazariboflavin.

The potentially solvent-dependent influence of the methylation pattern on the proton hyperfine interaction is established by comparing several photo-CIDNP experiments in DMSO-d_6_ and D_2_O. A comparable effect was reported for FMN derivatives, although only the 7α-methyl group showed an impact on the hyperfine coupling constant ratio of positions 6 and 8 [29]. Replacing N5 with C5-H, and thus increasing the absolute spin density of nuclei in the 5-deazaisoalloxazine moiety, seems to enforce the dependence of the proton hyperfine couplings of the xylene moiety on the methylation pattern. Furthermore, the hyperfine coupling of position 7 in 5-deazariboflavin derivatives is systematically stronger than that in the respective demethylated FMN derivatives. No CIDNP signal was reported for this position in the neutral radical species of FMN, 7-demethyl-FMN and 7,8-didemethyl-FMN or in the anionic radical species of 7-demethyl-FMN. All other FMN derivatives showed weak CIDNP signals, while all 5-deazariboflavin derivatives yielded a more substantial CIDNP polarization independently of the protonation state. This is in accordance with the calculated spin densities of riboflavin and 5-deazariboflavin derivatives, as, for the latter, a significantly higher spin density is found for protons H7 and H7α.

## 4. Materials and Methods

### 4.1. Sample Preparation

l-Tryptophan and D_2_O (99.9%) were purchased from Sigma-Aldrich, Saint Louis, MO, USA. NaOD (40% *w*/*w*, 99.5% D) and DCl (22% *w*/*w*, 99.5% D) were purchased from Cambridge Isotope Laboratories, Tewksbury, MA, USA. DMSO-d_6_ was purchased from Deutero GmbH, Kastellaun, Germany. l-Tyrosine was purchased from Carl Roth GmbH + Co. KG, Karlsruhe, Germany. These chemicals were used without further purification. 5-Deazariboflavin, 7-demethyl-5-deazariboflavin, 8-demethyl-5-deazariboflavin and 7,8-didemethyl-5-deazariboflavin were synthesized by following and adapting a procedure described in [11,29]. 5-Deazariboflavin, 7-demethyl-5-deazariboflavin, 8-demethyl-5-deazariboflavin and 7,8-didemethyl-5-deazariboflavin were purified by HPLC (LiChrospher RP-18 column, 18 mm × 20 mm) by using a linear 12–30% gradient of methanol in water (flow rate: 10 mL/min; retention time: 18 min). UV-VIS and NMR samples were prepared by dissolving the respective 5-deazariboflavin compound in DMSO-d_6_ or D_2_O, respectively. The used concentration is given with the respective experiment. For CIDNP samples dissolved in D_2_O or DMSO-d_6_, the concentrations of the 5-deazariboflavin derivative and l-tryptophan or l-tyrosine are given with the respective experiment. The pH values of aqueous samples were adjusted by adding small amounts of DCl or NaOD.

### 4.2. UV-VIS, NMR and Photo-CIDNP Spectroscopy

For UV-VIS measurements, a UV-2450 spectrometer manufactured by Shimadzu (Shimadzu Deutschland GmbH, Duisburg, Germany), as well as Hellma cuvettes of the type 105.250-QS (Hellma GmbH & Co. KG, Müllheim, Germany), were used.

NMR and photo-CIDNP experiments were performed as previously described [24]. The wavelength and pulse energy used for illumination during CIDNP experiments are given with the respective experiment. All experimental data were acquired at 293 K.

### 4.3. Density Functional Theory Calculations

The ORCA program package (ORCA versions 3.0 [58] and 5.0 [59]) was used for DFT calculations. The input structures of demethylated 5-deazariboflavins and riboflavins were generated and preliminarily optimized using Avogadro (Avogadro version 1.2.0) [60]. Structures were optimized by using the B3LYP functional [61] and the TZVP basis set [62], as well as the def2/J auxiliary basis [63]. The B3LYP functional and the EPR-II basis set [64] were used for subsequent calculations of hyperfine coupling constants and *g* factors. A CPCM model [65] implemented in the ORCA program package was used for the simulation of water or DMSO solvation.

## 5. Conclusions

The incorporation of modified flavin cofactors is a key approach to understanding and altering the reaction mechanisms of flavoproteins. Nor analogs of 5-deazariboflavin are proposed as potential alternatives for cofactor replacement experiments. This photo-CIDNP study of demethylated 5-deazariboflavins gives a thorough understanding of the proton hyperfine structures of 5-deazariboflavin radicals, particularly with respect to the influence of the methyl groups. Furthermore, when compared to demethylated FMN derivatives, it provides deeper insights into the impact of N5 on the flavin’s electronic structure.

## Figures and Tables

**Table 1:** Isotropic *g* factors of the demethylated 5-deazariboflavin radicals calculated using density functional theory (DFT). All calculations were carried out using a conductor-like polarizable continuum model (CPCM) to simulate DMSO solvation.

Derivative	*g*_iso_	
	5-Deazariboflavin^●−^	5-Deazariboflavin(H1)^●^
5-Deazariboflavin	2.00281	2.00275
7-Demethyl-5-deazariboflavin	2.00280	2.00274
8-Demethyl-5-deazariboflavin	2.00282	2.00276
7,8-Didemethyl-5-deazariboflavin	2.00283	2.00276

**Table 2:** Isotropic hyperfine coupling constants *A*_iso_ of 5-deazariboflavin radicals and demethylated derivatives in DMSO-d_6_. Only protons of the 5-deazaisoalloxazine moiety are listed. The proton CIDNP intensities and hyperfine couplings of methyl groups are averaged, as rotation is expected to be fast enough for thermal averaging. Values are normalized with respect to H5. The relative CIDNP intensity of H7 in 7,8-didemethyl-5-deazariboflavin is not given, as the poor signal-to-noise ratio did not permit a proper fit. The values from DFT calculations are given as absolute values in MHz as well as relative values. The relative values are multiplied by −1 for better comparability with the relative CIDNP intensities.

		5-Deazariboflavin^●−^		5-Deazariboflavin(H1)^●^		
		Abs. *A*_iso_/MHz (DFT)	Rel. *A*_iso_ (DFT)	Abs. *A*_iso_/MHz (DFT)	Rel. *A*_iso_ (DFT)	Rel. *A*_iso_ (CIDNP)
5-Deazariboflavin	H5	−34.26	1.00	−37.48	1.00	1.00
	H6	−12.29	0.36	−11.83	0.32	0.31
	H7α	−4.63	0.14	−4.45	0.12	0.11
	H8α	14.75	−0.43	14.80	−0.39	−0.41
	H9	3.00	−0.09	3.57	−0.10	−0.05
7-Demethyl- 5-deazariboflavin	H5	−34.12	1.00	−37.44	1.00	1.00
	H6	−14.05	0.41	−13.42	0.36	0.37
	H7	4.21	−0.12	4.04	−0.11	−0.14
	H8α	14.86	−0.44	14.72	−0.39	−0.37
	H9	2.63	−0.08	3.28	−0.09	−0.08
8-Demethyl- 5-deazariboflavin	H5	−33.16	1.00	−36.78	1.00	1.00
	H6	−12.54	0.38	−12.24	0.33	0.41
	H7α	−4.63	0.14	−4.47	0.12	0.08
	H8	−15.28	0.46	−14.99	0.41	0.53
	H9	2.28	−0.07	3.06	−0.08	−0.03
7,8-Didemethyl- 5-deazariboflavin	H5	−33.39	1.00	−37.02	1.00	1.00
	H6	−13.46	0.40	−13.10	0.35	0.32
	H7	4.66	−0.14	4.56	−0.12	–
	H8	−14.62	0.44	−14.38	0.39	0.41
	H9	2.05	−0.06	2.86	−0.08	−0.04

## Data Availability

The raw data that support the findings of this study are available from the corresponding author upon reasonable request.

## Supplementary Materials

The following supporting information can be downloaded at: https://www.mdpi.com/article/10.3390/ijms25020848/s1.
