# Supplementary material for: Shining a Spotlight on Methyl Groups: Photochemically Induced Dynamic Nuclear Polarization Spectroscopy of 5-Deazariboflavin and Its Nor Analogs
Source: Int J Mol Sci. 2024 Jan 10;25(2):848. doi: 10.3390/ijms25020848 (PMC10815406; doi:10.3390/ijms25020848)
Supplement: Supplementary file 1 [file ijms-25-00848-s001.zip › ijms-2789816-supplementary.pdf]

Supporting Information

Shining a Spotlight on Methyl Groups: Photochemically Induced Dynamic Nuclear Polarization Spectroscopy of 5-Deazariboflavin and its Nor Analogs

Sabrina Panter[1], Audrey Ayekoi[1], Jannis Tesche[1], Jing Chen[1], Boris Illarionov[1], Adelbert Bacher[3], Markus Fischer[2] and Stefan Weber[1]

[1] Institut für Physikalische Chemie, Albert-Ludwigs-Universität Freiburg, 79104 Freiburg, Germany

[2] Institut für Lebensmittelchemie, Universität Hamburg, Grindelallee 117, 20146 Hamburg, Germany

[3] TUM School of Natural Sciences, Technische Universität München, 85747 Garching, Germany

Contents

1 Synthesis of Demethylated 5-Deazariboflavin Derivatives 2

1.1 General Information 2

1.2 Spectroscopic Data of 5-Deazariboflavin and 5-Deazariboflavin Analogs 3

2 Absorption Data of Demethylated 5-Deazariboflavin Derivatives 11

3 DFT Calculations of Electronic Properties for Demethylated 5-Deazariboflavin Radicals 12

4 Photo-CIDNP Experiments of Demethylated 5-Deazariboflavin Derivatives in D_2O 14

5 Correlation of Photo-CIDNP Intensities in Aqueous Solution 17

6 Mulliken Spin Densities of Demethylated 5-Deazariboflavin and Riboflavin Derivatives **22**

References **26**

1 Synthesis of Demethylated 5-Deazariboflavin Derivatives

1.1 General Information

5-Deazariboflavin, 7-demethyl-5-deazariboflavin, 8-demethyl-5-deazariboflavin and 7,8-didemethyl-5-deazariboflavin were synthesized following and adapting published procedures [1, 2]. The synthetic route to prepare 8-demethyl-5-deazariboflavin is shown exemplary in Figure S1. All chemicals used for the synthesis of 5-deazaflavin and the demethylated 5-deazariboflavin derivatives were purchased from commercial sources and used as received. Reactions were followed using thin layer chromatography on silica gel-coated plates (Merck 60 F254). High Resolution Mass Spectra (HRMS) were measured on an Advance or exactive instrument (Thermo Scientific, Waltham, MA), equipped with an atmospheric pressure chemical ionization (APCI) or electrospray ionization (ESI) source in the positive-ion mode. ^{1}H NMR experiments were performed on a Bruker Avance II HD 400 MHz NMR spectrometer or a Bruker Avance III HD 500 MHz NMR spectrometer. ^{13}C NMR experiments were performed on a Bruker Avance III HD 600 MHz spectrometer. Chemical shifts are reported in ppm using the respective solvent as internal standard (DMSO-d$_6$: δ(H) = 2.50 ppm, δ(C) = 39.52 ppm). Data are reported as: s (singulet) and m (multiplet).

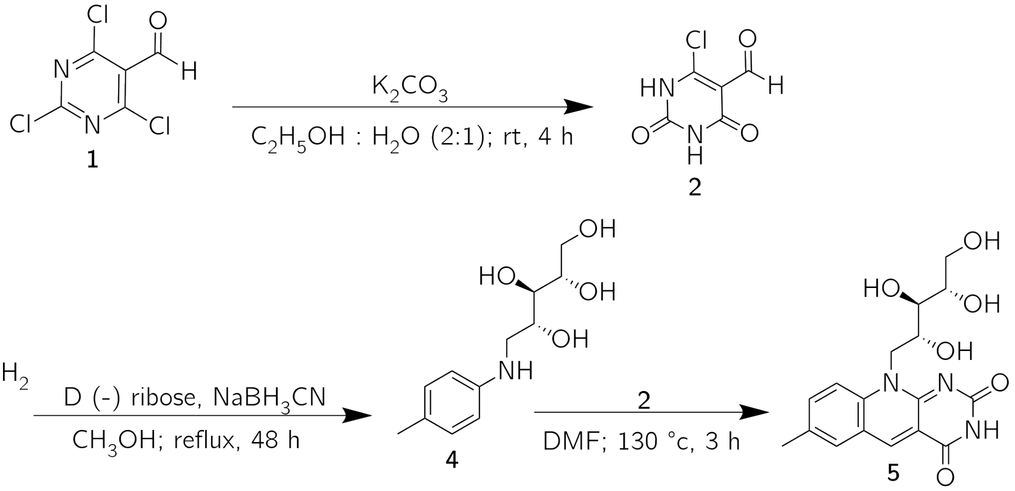

Figure S1 Synthesis of 8-demethyl-5-deazariboflavin according and following procedures described in [1, 2]. This route was adapted for synthesis of 5-deazariboflavin, 7-demethyl-5-deazariboflavin and 7,8-didemethyl-5-deazariboflavin, as well.

1.2 Spectroscopic Data of 5-Deazariboflavin and 5-Deazariboflavin Analogs

5-Deazariboflavin

^{1}H NMR (DMSO-d$_6$, 500 MHz): δ / ppm = 11.04 (s, 1H), 8.86 (s, 1H), 7.96 (s, 1H), 7.87 (s, 1H), 5.15–5.14 (m, 1H), 5.01–5.00 (m, 1H), 4.91–4.90 (m, 1H), 4.82–4.81 (m, 1H), 4.67–4.64 (m, 1H), 4.51–4.49 (m, 1 H), 4.25–4.22 (m, 1H), 3.68–3.60 (m, 3H), 3.49–3.45 (m, 1H), 2.46 (s, 3H), 2.35 (s, 3H). **^{13}C NMR** (DMSO-d$_6$, 500 MHz): δ / ppm = 162.17, 157.60, 156.39, 156.35, 145.99, 141.00, 139.83, 133.61, 130.56, 119.64, 117.89, 113.71, 73.72, 72.93, 69.61, 63.46, 20.93, 18.66. **HRMS (ESI)**: m/z for $C_{18}H_{22}O_6N_3$ [M$^+$H]$^+$ calculated: 376.1503, found: 376.1502.

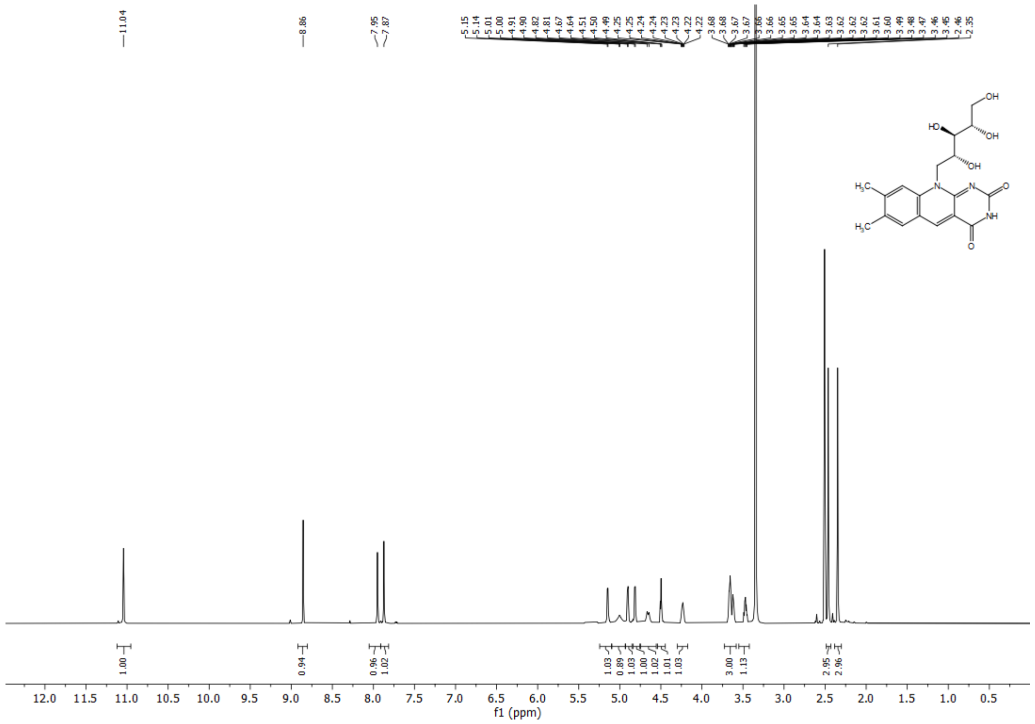

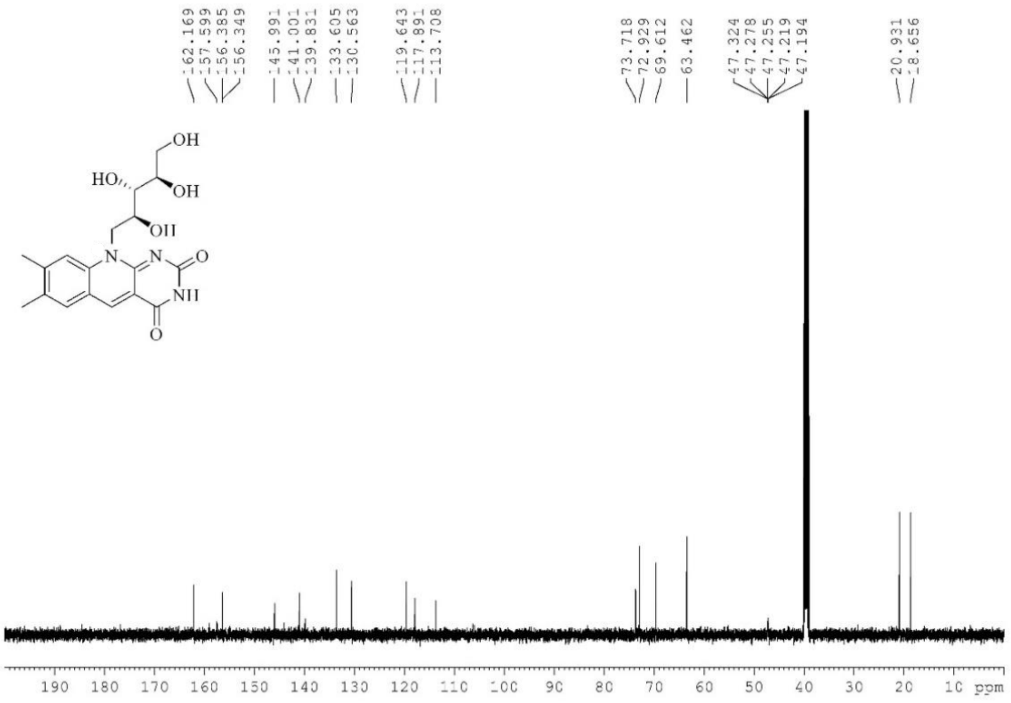
73.718
72.929
69.612
63.462
20.931
OH
HO
OH
OH
N
N
O
NH
O
190 180 170 160 150 140 130 120 90 80 70 60 50 40 30 20 ppm

7-Demethyl-5-deazariboflavin

^{1}H NMR (DMSO-d$_6$, 500 MHz): δ / ppm = 11.09 (s, 1H), 8.98 (s, 1H), 8.06–8.05 (m, 1H), 7.98(s, 1H), 7.39–7.36 (m, 1H), 5.15–5.13 (m, 1H), 5.00–4.97 (m, 1H), 4.90–4.89 (m, 1H), 4.84–4.83 (m, 1H), 4.68–4.66 (m, 1H), 4.51–4.48 (m, 1H), 4.26–4.22 (m, 1 H), 3.67–3.60 (m, 3H), 3.48–3.44 (m, 1H), 2.54 (s, 3H). **^{13}C NMR** (DMSO-d$_6$, 600 MHz): δ / ppm = 162.22, 158.01, 156.48, 146.25, 141.60, 141.51, 131.26, 125.94, 119.39, 117.54, 113.86, 73.73, 72.93, 69.50, 63.47, 47.26, 22.38. **HRMS (ESI)**: m/z for $C_{17}H_{20}O_6N_3$ $[M^{+}H]^{+}$ calculated: 362.1347, found: 362.1346.

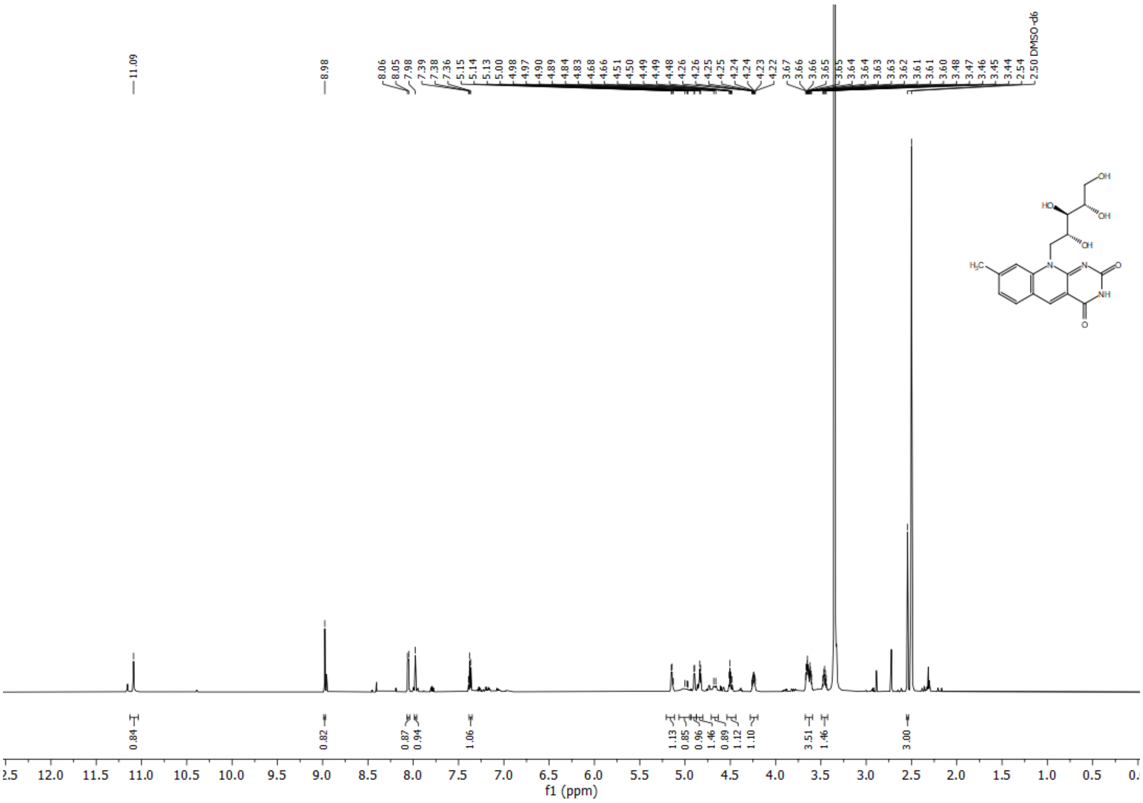

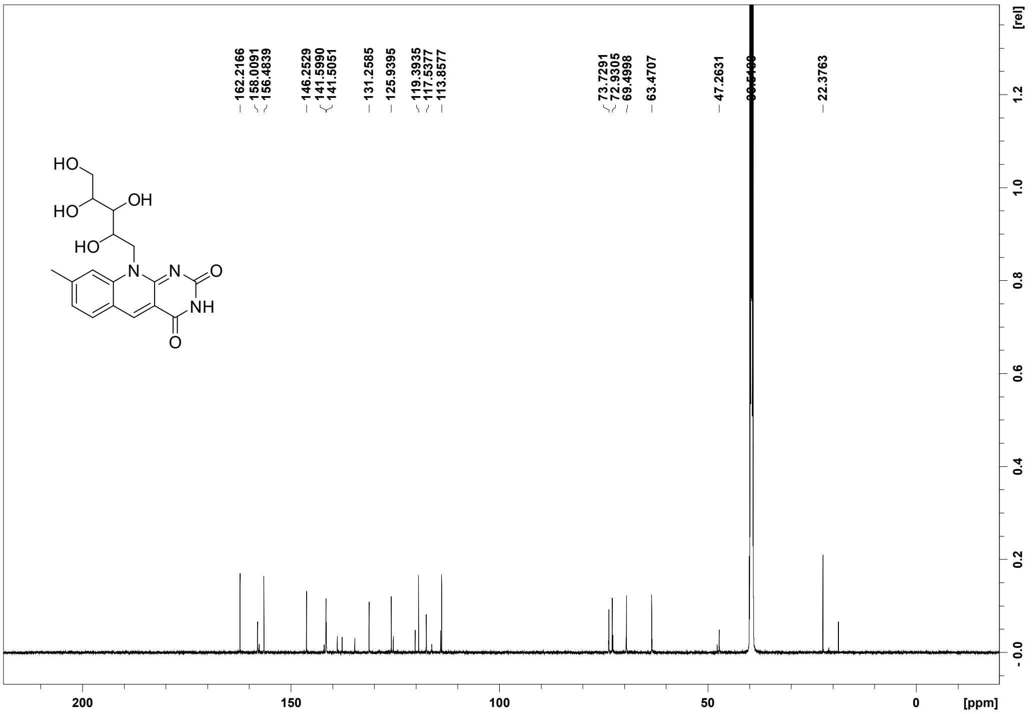

162.2166
158.0091
156.4839
146.2529
141.5990
141.5051
131.2585
125.9395
119.3935
117.5377
113.8577
73.7291
72.9305
69.4998
63.4707
47.2631
22.3763
HO
HO
OH
HO
N
N
O
NH
O
[rel]
1.2
1.0
0.8
0.6
0.4
0.2
0.0
200
150
100
50
0
[ppm]

8-Demethyl-5-deazariboflavin

^{1}H NMR (DMSO-d$_6$, 500 MHz): δ / ppm = 11.09 (s, 1H), 8.98 (s, 1H), 8.06–8.05 (m, 1H), 7.98(s, 1H), 7.39–7.36 (m, 1H), 5.15–5.13 (m, 1H), 5.00–4.97 (m, 1H), 4.90–4.89 (m, 1H), 4.84–4.83 (m, 1H), 4.68–4.66 (m, 1H), 4.51–4.48 (m, 1H), 4.26–4.22 (m, 1 H), 3.67–3.60 (m, 3H), 3.48–3.44 (m, 1H), 2.54 (s, 3H). **^{13}C NMR** (DMSO-d$_6$, 600 MHz): δ / ppm = 162.58, 150.03, 156.96, 141.99, 140.21, 136.94, 134.30, 130.88, 121.82, 118.40, 115.33, 74.30, 73.27, 70.12, 63.86, 48.11, 20.62. **HRMS (ESI)**: m/z for $C_{17}H_{20}O_6N_3$ $[M^{+}H]^{+}$ calculated: 362.1347, found: 362.1346.

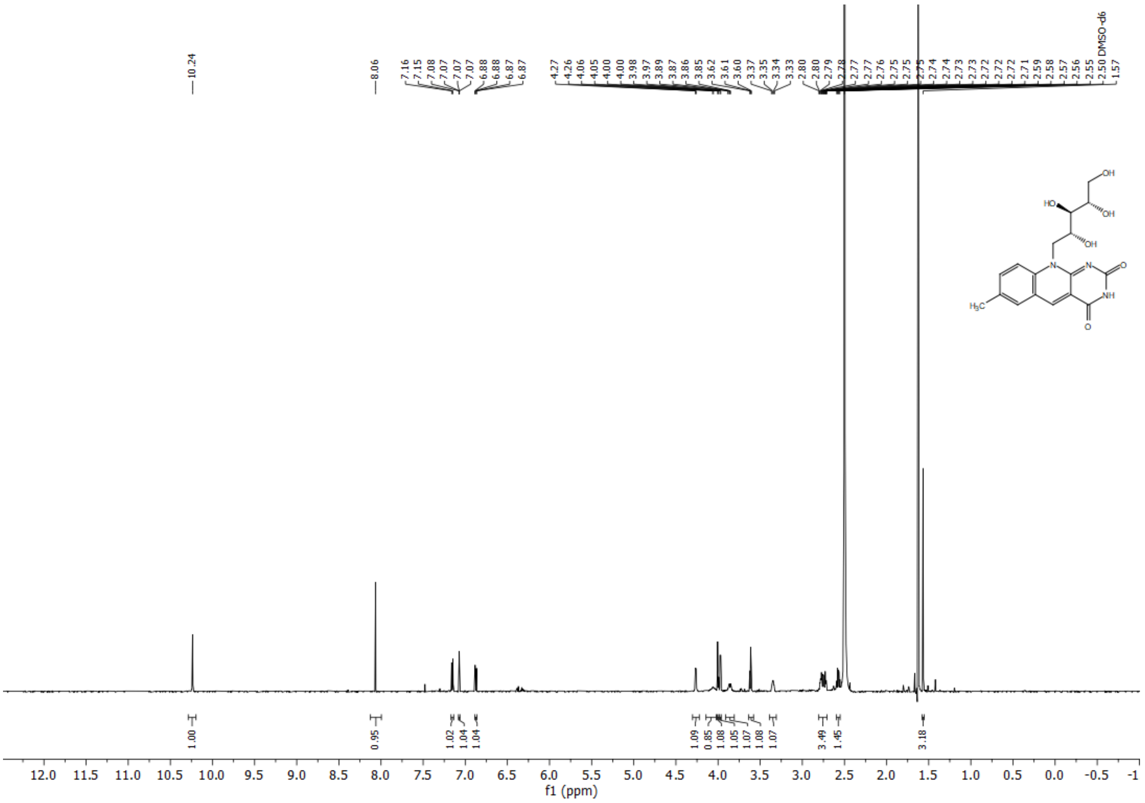

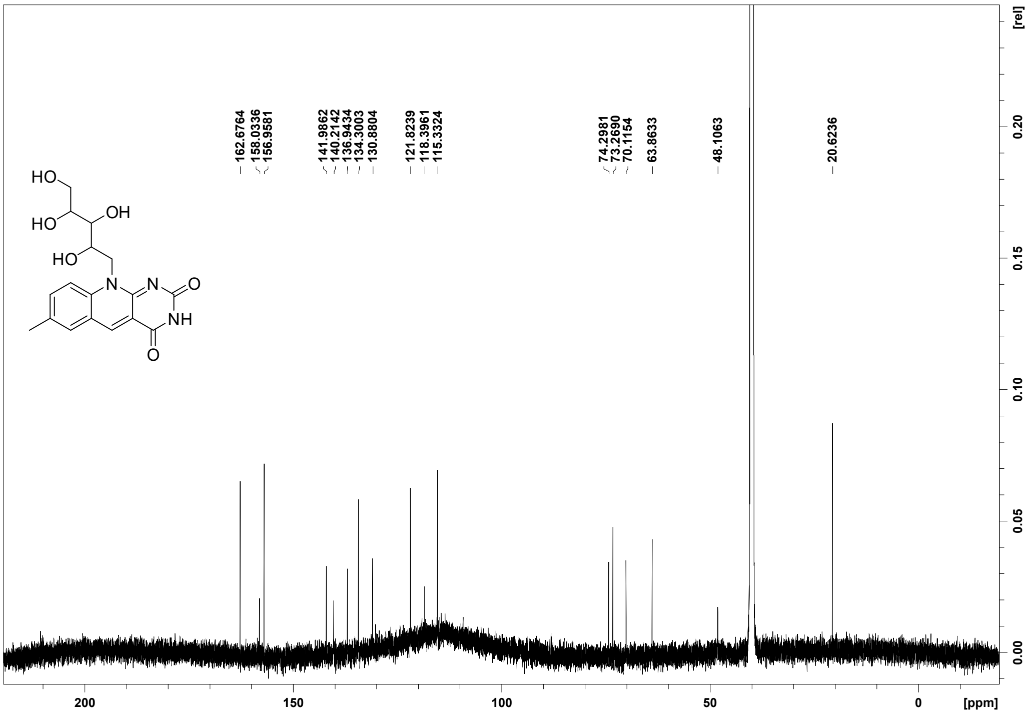
162.6764
158.0336
156.9581
141.9862
140.2142
136.9434
134.3003
130.8804
121.8239
118.3961
115.3324
74.2981
73.2690
70.1154
63.8633
48.1063
20.6236
[rel]
0.20
0.15
0.10
0.05
0.00
200
150
100
50
0
[ppm]
HO
HO
OH
HO
N
N
O
NH
O

7,8-Didemethyl-5-deazariboflavin

^{1}H NMR (DMSO-d$_6$, 400 MHz): δ / ppm = 11.13 (s, 1H), 9.03 (s, 1H), 8.18–8.17 (m, 1H), 8.13–8.12 (m, 1H), 7.92–7.89 (m, 1H), 7.53–7.51 (m, 1H), 5.13–5.12 (m, 1H), 4.97 (s, 1H), 4.88–4.87 (m, 1H), 4.83–8.82 (m, 1H), 4.76–4.73 (m, 1H), 4.48–4.46 (m, 1H), 4.24 (s, 1H), 3.66–3.60 (m, 3H), 3.47–3.44 (m, 1H). **^{13}C NMR** (DMSO-d$_6$, 600 MHz): δ / ppm = 162.56, 158.38, 156.91, 142.31, 141.90, 135.27, 131.89, 124.68, 121.74, 118.42, 115.38, 74.23, 73.22, 70.00, 63.84, 48.02.

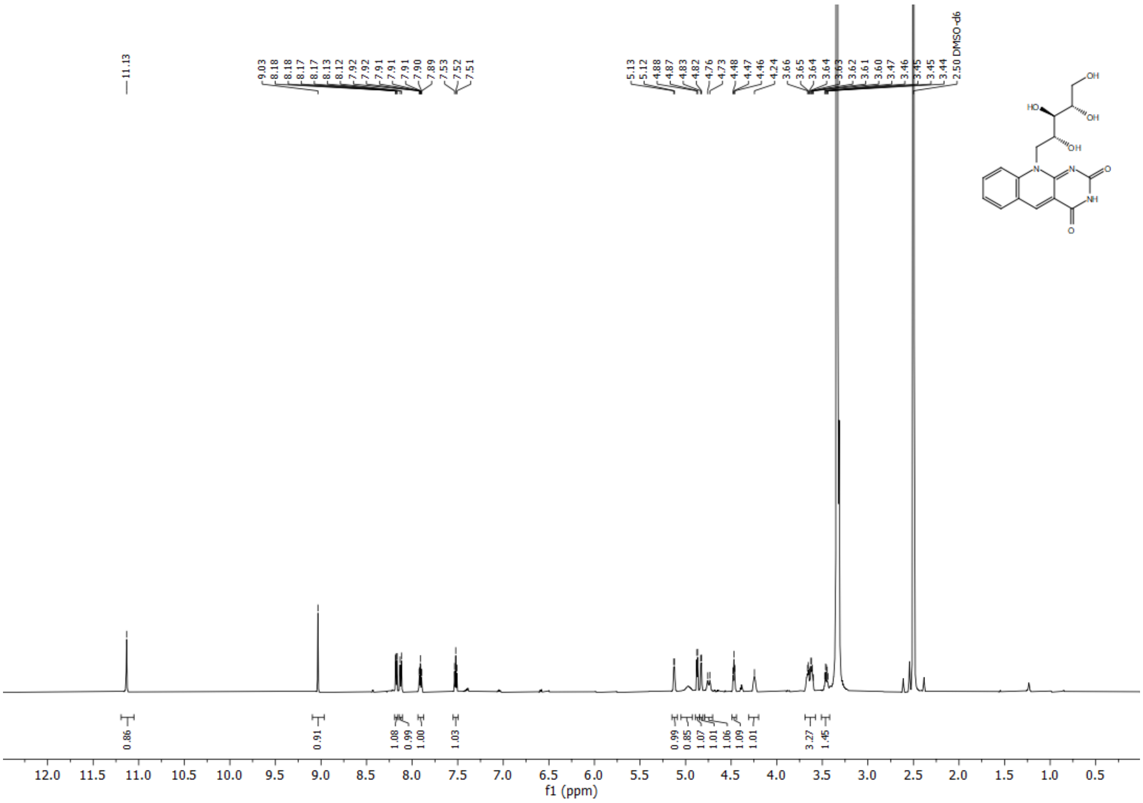

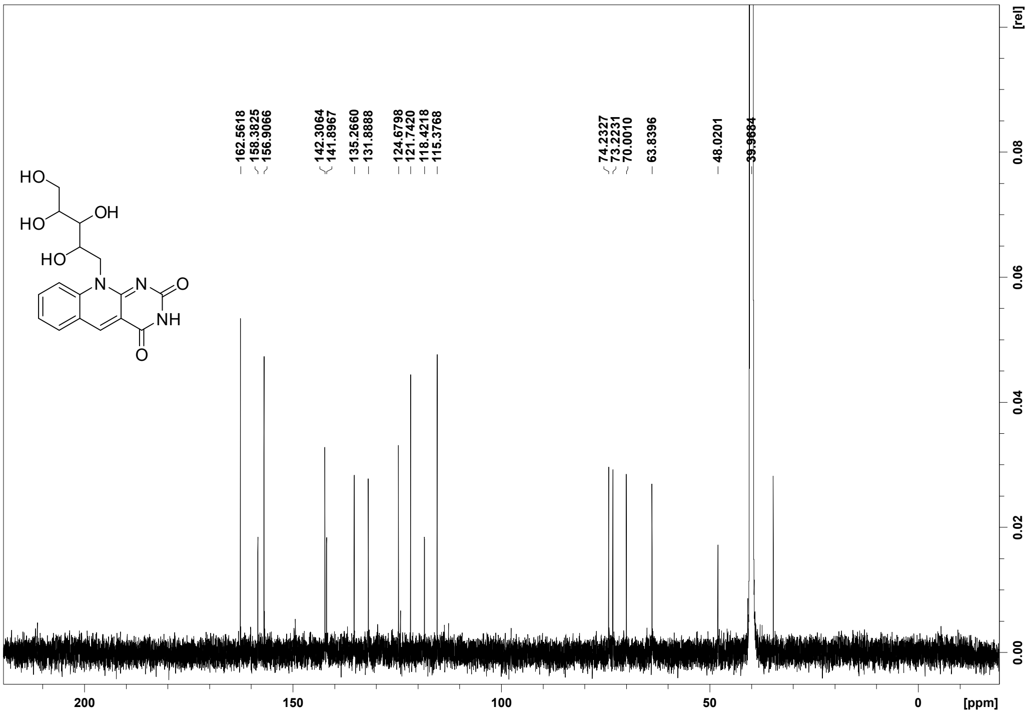
162.5618
158.3825
156.9066
142.3064
141.8967
135.2660
131.8888
124.6798
121.7420
118.4218
115.3768
74.2327
73.2231
70.0010
63.8396
48.0201
HO
HO
OH
HO
N
N
O
NH
O
[rel]
0.08
0.06
0.04
0.02
0.00
200
150
100
50
0
[ppm]

2 Absorption Data of Demethylated 5-Deazariboflavin Derivatives

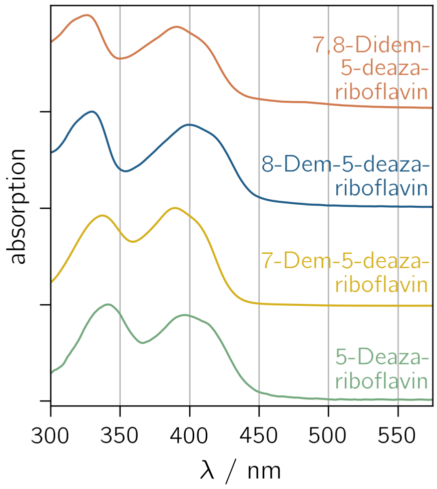

Figure S2 Normalized absorption spectra of 5-deazariboflavin, 7-demethyl-5-deazariboflavin, 8-demethyl-5-deazariboflavin and 7,8-didemethyl-5-deazariboflavin in H_2O. "Demethyl" is abbreviated by "dem".

Table S1 Absorption maxima of 5-deazariboflavin and demethylated 5-deazariboflavin derivatives in H_2O.

	$\lambda_{max,1}$ / nm	$\lambda_{max,2}$ / nm
5-Deazariboflavin	342	398
7-Demethyl-5-deazariboflavin	338	390
8-Demethyl-5-deazariboflavin	329	402
7,8-Didemethyl-5-deazariboflavin	327	391

3 DFT Calculations of Electronic Properties for Demethylated 5-Deazariboflavin Radicals

Table S2 Calculated isotropic hyperfine coupling constants A_{iso} of H1 and H3 of 5-deazariboflavin radicals and demethylated derivatives. All calculations were carried out using a conductor-like polarizable continuum model to simulate DMSO solvation. The values are given as absolute values in MHz.

	A_{iso} / MHz H3	H1
5-Deazariboflavin(H1)$^{\bullet}$	0.0454	0.3534
5-Deazariboflavin$^{\bullet-}$	−0.1434	–
7-Demethyl-5-deazariboflavin(H1)$^{\bullet}$	0.4155	−0.0415
7-Demethyl-5-deazariboflavin$^{\bullet-}$	0.0504	–
8-Demethyl-5-deazariboflavin(H1)$^{\bullet}$	0.2857	0.0227
8-Demethyl-5-deazariboflavin$^{\bullet-}$	−0.1136	–
7,8-Didemethyl-5-deazariboflavin(H1)$^{\bullet}$	0.3369	−0.0386
7,8-Didemethyl-5-deazariboflavin$^{\bullet-}$	−0.1398	–

Table S3 Isotropic g factors g_{iso} of demethylated 5-deazariboflavin radicals calculated using DFT. All calculations were carried out using a conductor-like polarizable continuum model to simulate water solvation.

	g_{iso} 5-Deazariboflavin$^{\bullet-}$	5-Deazariboflavin(H1)$^{\bullet}$
5-Deazariboflavin	2.002 80	2.002 76
7-Demethyl-5-deazariboflavin	2.002 80	2.002 75
8-Demethyl-5-deazariboflavin	2.002 82	2.002 77
7,8-Didemethyl-5-deazariboflavin	2.002 82	2.002 77

Table S4 Isotropic hyperfine coupling constants A_{iso} of 5-deazariboflavin radicals and demethylated derivatives in D_2O. "Demethyl" is abbreviated "dem". Relative CIDNP intensities are taken from Figures S4 and S5. The relative CIDNP intensities of H6 and H9 in 7,8-didemethyl-5-deazariboflavin are not given due to signal overlap. The proton CIDNP intensities and hyperfine couplings of methyl groups are averaged as rotation is expected to be fast enough for thermal averaging. Values are normalized with respect to H5. The values from DFT calculations are given as absolute values in MHz as well as relative values.

		5-Deazariboflavin$^{\bullet -}$			5-Deazariboflavin(H1)$^{\bullet}$		
		abs. A_{iso} / MHz (DFT)	rel. A_{iso} (DFT)	rel. A_{iso} (CIDNP)	abs. A_{iso} / MHz (DFT)	rel. A_{iso} (DFT)	rel. A_{iso} (CIDNP)
5-Deaza-riboflavin	H5	−34.54	1.00	1.00	−37.51	1.00	1.00
	H6	−12.50	0.36	0.32	−11.80	0.31	0.30
	H7α	−4.70	0.14	0.09	−4.45	0.12	0.08
	H8α	15.10	−0.44	−0.42	14.79	−0.39	−0.39
	H9	3.09	−0.09	−0.06	3.57	−0.10	−0.07
7-Dem-5-deaza-riboflavin	H5	−34.48	1.00	1.00	−37.57	1.00	1.00
	H6	−13.82	0.40	0.34	−13.14	0.35	0.39
	H7	4.17	−0.12	−0.15	4.01	−0.11	−0.13
	H8α	14.73	−0.43	−0.42	14.52	−0.39	−0.41
	H9	2.68	−0.08	−0.12	3.32	−0.09	−0.21
8-Dem-5-deaza-riboflavin	H5	−33.52	1.00	1.00	−36.74	1.00	1.00
	H6	−12.39	0.37	0.36	−12.10	0.33	0.38
	H7α	−4.66	0.14	0.10	−4.55	0.12	0.09
	H8	−15.29	0.46	0.41	−15.10	0.41	0.35
	H9	2.38	−0.07	−0.07	3.10	−0.08	−0.12
7,8-Didem-5-deaza-riboflavin	H5	−33.93	1.00	1.00	−37.11	1.00	1.00
	H6	−13.44	0.40	–	−13.00	0.35	–
	H7	4.66	−0.14	−0.13	4.52	−0.12	−0.12
	H8	−4.64	0.43	0.39	−14.28	0.38	0.47
	H9	2.22	−0.07	–	2.85	−0.08	–

4 Photo-CIDNP Experiments of Demethylated 5-Deazariboflavin Derivatives in D_2O

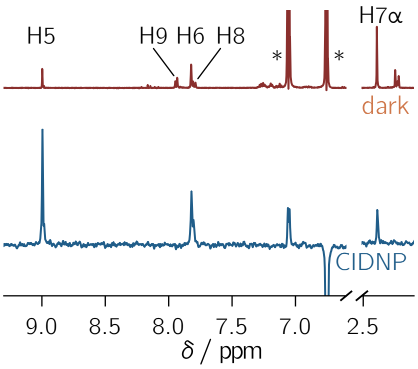

Figure S3 Thermal (red) and transient ^1H photo-CIDNP (blue) spectra of 8-demethyl-5-deazariboflavin (0.25 mM) and L-tyrosine (1.40 mM) at pH 1.7 in D_2O. Only the ^1H resonances of the 5-deazaisoalloxazine moiety are shown. Resonances of L-tyrosine are denoted with an asterisk. The sample was irradiated at 420 nm with pulse energies of 6.0 mJ. All spectra were referenced to the resonance of HDO at 4.7 ppm.

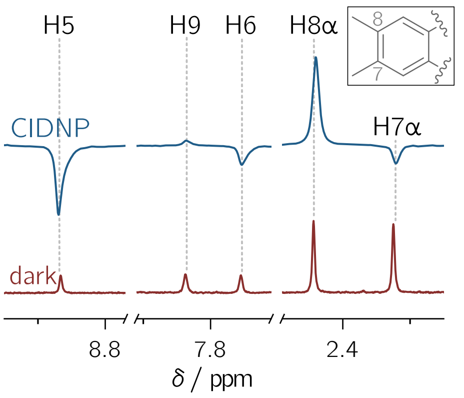

(a) L-Tryptophan was added to a final concentration of 1.4 mM and 3.0 mM, respectively. The pH was adjusted to 1.8. A pulse energy of 4.6 mJ was used for illumination.

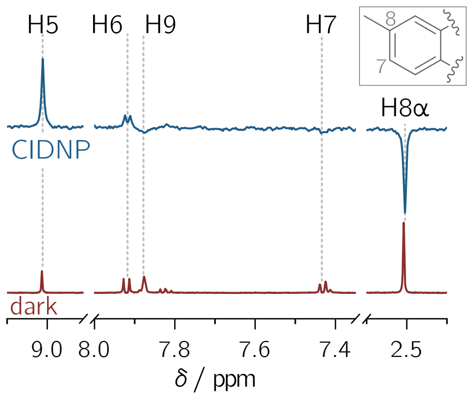

(b) L-Tyrosine was added to a final concentration of 1.4 mM. The pH was adjusted to 1.7. A pulse energy of 5.6 mJ was used for illumination.

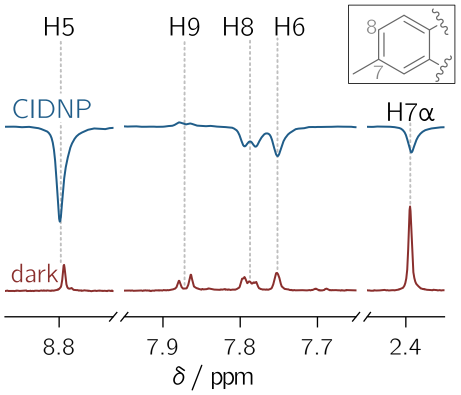

(c) L-Tryptophan was added to a final concentration of 5.0 mM. The pH was adjusted to 1.5. A pulse energy of 4.9 mJ was used for illumination.

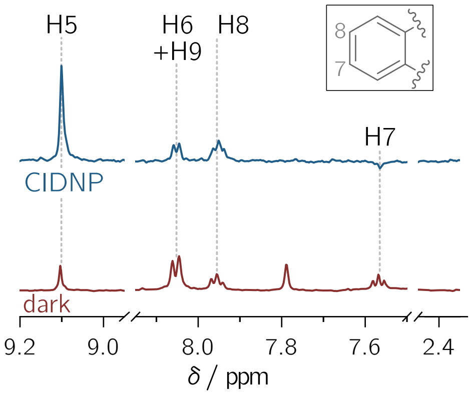

(d) L-Tyrosine was added to a final concentration of 0.9 mM. The pH was adjusted to 1.7. A pulse energy of 9.1 mJ was used for illumination.

Figure S4 Thermal (red) and transient ^{1}H photo-CIDNP (blue) spectra of (a) 5-deazariboflavin, (b) 7-demethyl-5-deazariboflavin, (c) 8-demethyl-5-deazariboflavin and (d) 7,8-didemethyl-5-deazariboflavin in D_2O at acidic pH. Only the ^{1}H resonances of the 5-deazaisoalloxazine moiety are shown. 5-Deazariboriboflavin derivatives were added to a final concentration of 0.25 mM if not indicated otherwise. The electron donor moiety and the pH of the sample are indicated with the respective experiment. All samples were irradiated at 420 nm. All spectra were referenced to the resonance of HDO at 4.7 ppm.

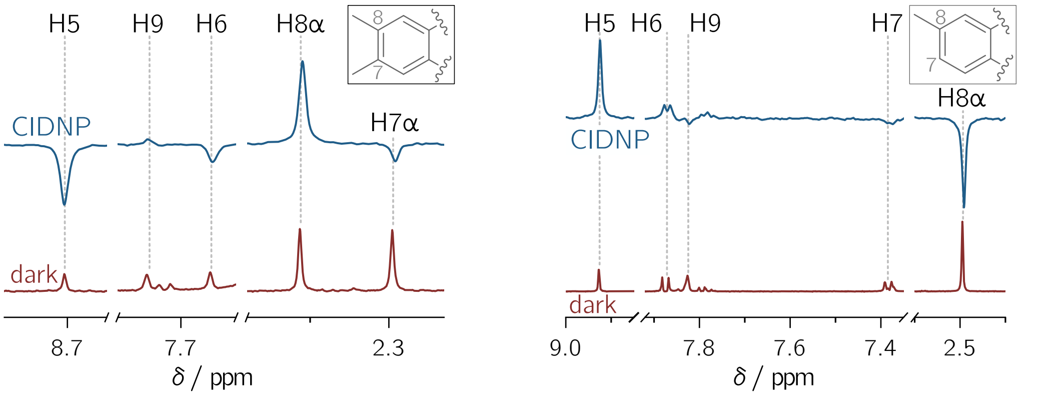

(a) 5-Deazariboflavin and L-tryptophan were added to a final concentration of 1.4 mM and 3.0 mM, respectively. The pH was adjusted to 8.5. A pulse energy of 4.6 mJ was used for illumination.

(b) L-Tyrosine was added to a final concentration of 1.3 mM. The pH was adjusted to 8.6. A pulse energy of 6.0 mJ was used for illumination.

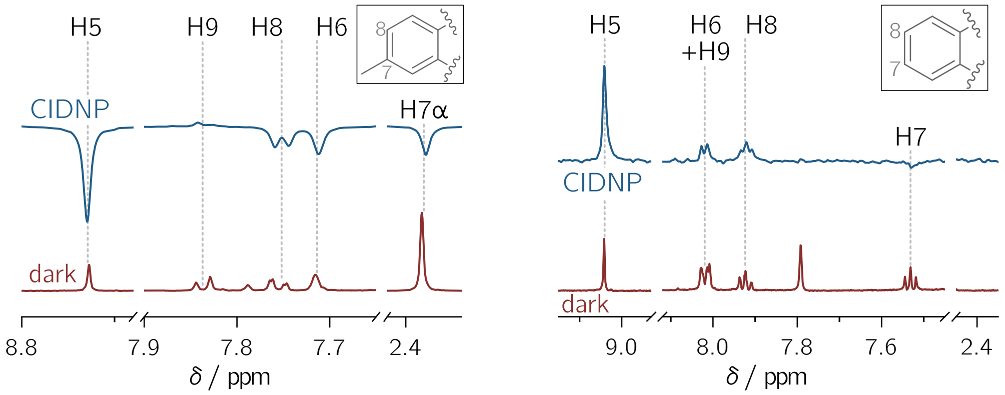

(c) L-Tryptophan was added to a final concentration of 5.0 mM. The pH was adjusted to 7.6. A pulse energy of 5.6 mJ was used for illumination.

(d) L-Tyrosine was added to a final concentration of 0.9 mM. The pH was adjusted to 7.8. A pulse energy of 5.6 mJ was used for illumination.

Figure S5 Thermal (red) and transient ^{1}H photo-CIDNP (blue) spectra of (a) 5-deazariboflavin, (b) 7-demethyl-5-deazariboflavin, (c) 8-demethyl-5-deazariboflavin and (d) 7,8-didemethyl-5-deazariboflavin in D_2O at basic pH. Only the ^{1}H resonances of the 5-deazaisoalloxazine moiety are shown. 5-Deazariboflavin derivatives were added to a final concentration of 0.25 mM if not indicated otherwise. The electron donor moiety and the pH of the sample are indicated with the respective experiment. All samples were irradiated at 420 nm. All spectra were referenced to the resonance of HDO at 4.7 ppm.

5 Correlation of Photo-CIDNP Intensities in Aqueous Solution

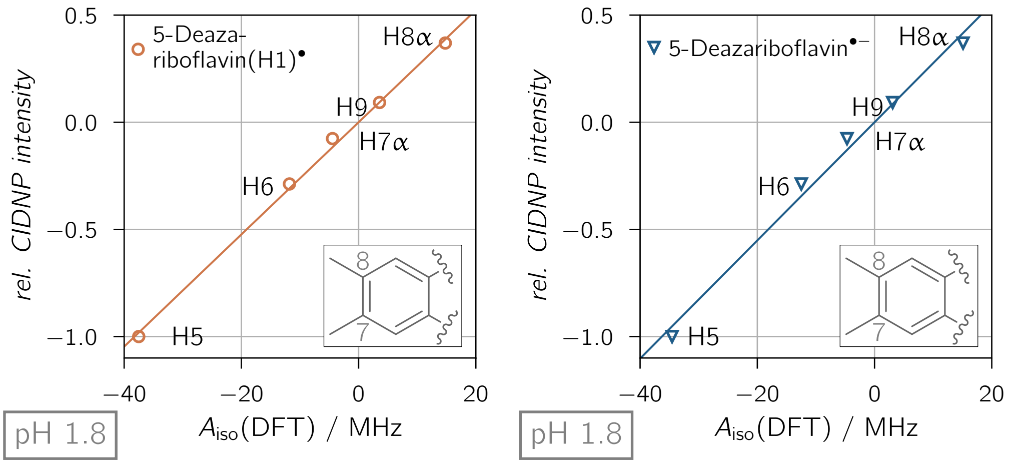

(a) 5-Deazariboflavin(H1)•: $m = 0.0262\,\text{MHz}^{-1}$, $R^2 = 0.9975$;
5-Deazariboflavin•−: $m = 0.0276\,\text{MHz}^{-1}$, $R^2 = 0.9902$.

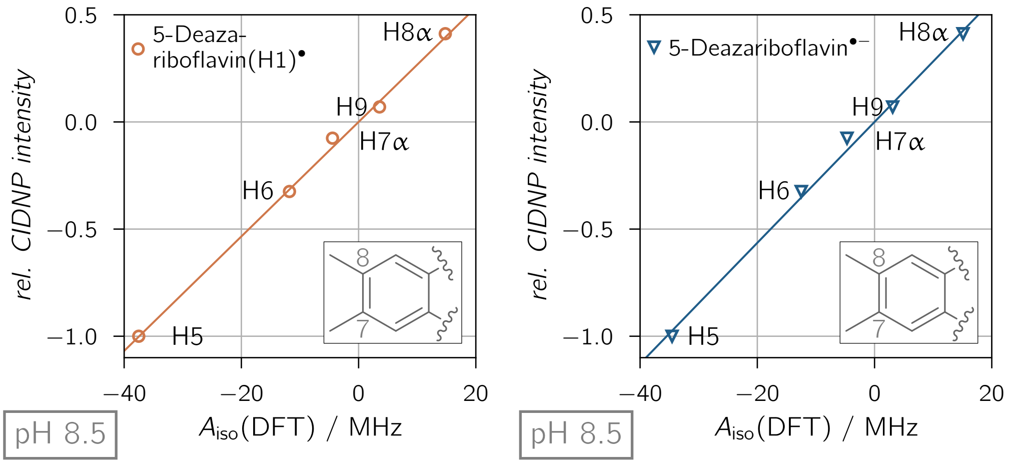

(b) 5-Deazariboflavin(H1)•: $m = 0.0267\,\text{MHz}^{-1}$, $R^2 = 0.9974$;
5-Deazariboflavin•−: $m = 0.0282\,\text{MHz}^{-1}$, $R^2 = 0.9954$.

Figure S6 Correlation plots of ^{1}H transient CIDNP experiments of 5-deazriboflavin in D_2O at a) pH 1.8 and b) pH 8.5. Linear fits were forced to go through the origin. Values for relative CIDNP intensities of a) are extracted from Figure S4 a) by fitting a Voigt line shape to the CIDNP signal. Values for relative CIDNP intensities of b) are extracted from Figure S5 a) by fitting a Voigt line shape to the CIDNP signals of H5, H7α and H8α and by fitting a Lorentzian function to the CIDNP signals of H6 and H9. A_{iso} from DFT calculations are listed in Table S4.

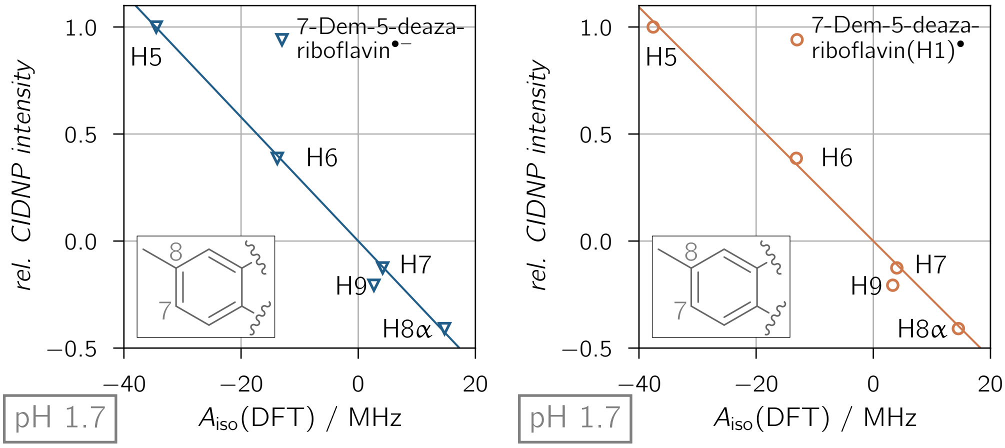

(a) 7-Demethyl-5-deazariboflavin(H1)$^{\bullet}$: $m = -0.0273\,\mathrm{MHz}^{-1}$, $R^2 = 0.9882$; 7-Demethyl-5-deazariboflavin$^{\bullet-}$: $m = -0.0289\,\mathrm{MHz}^{-1}$, $R^2 = 0.9867$.

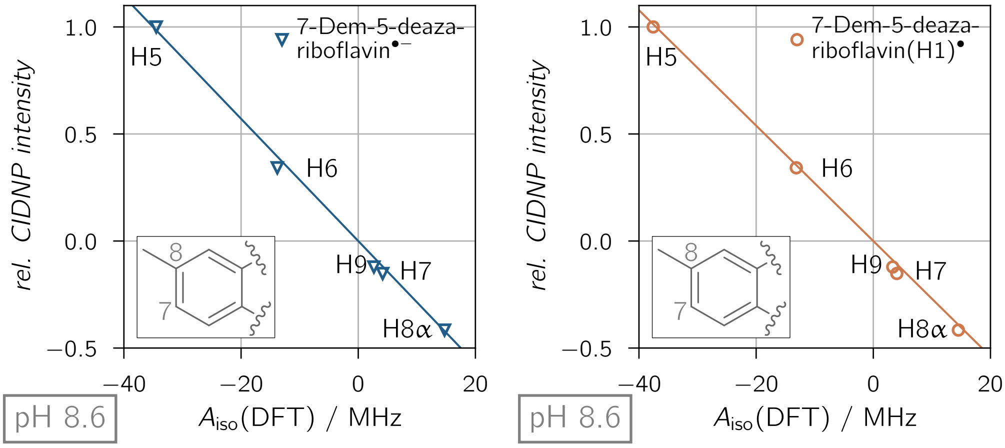

(b) 7-Demethyl-5-deazariboflavin(H1)$^{\bullet}$: $m = -0.0270\,\mathrm{MHz}^{-1}$, $R^2 = 0.9969$; 7-Demethyl-5-deazariboflavin$^{\bullet-}$: $m = -0.0286\,\mathrm{MHz}^{-1}$, $R^2 = 0.9951$.

Figure S7 Correlation plots of ^{1}H transient CIDNP experiments of 7-demethyl-5-deazriboflavin (7-dem-5-deazariboflavin) in D_2O at a) pH 1.7 and b) pH 8.6. Linear fits were forced to go through the origin. Values for relative CIDNP intensities were extracted from Figure S4 b) and Figure S5 b) by fitting a Voigt line shape to the CIDNP signals of H5 and H8α and by fitting a Lorentzian function to the CIDNP signals of H6, H7 and H9. A_{iso} from DFT calculations are listed in Table S4.

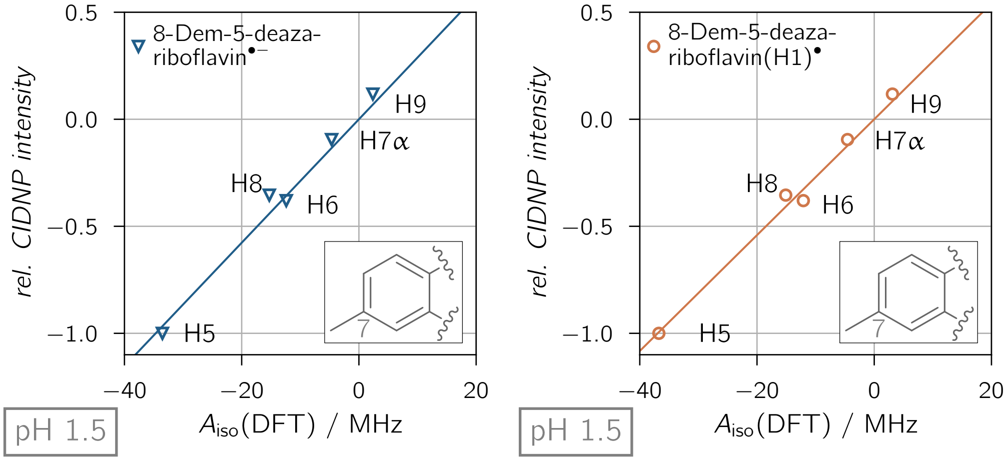

(a) 8-Demethyl-5-deazariboflavin(H1)•: $m = 0.0271\,\mathrm{MHz}^{-1}$, $R^2 = 0.9891$;
8-Demethyl-5-deazariboflavin•−: $m = 0.0288\,\mathrm{MHz}^{-1}$, $R^2 = 0.9815$.

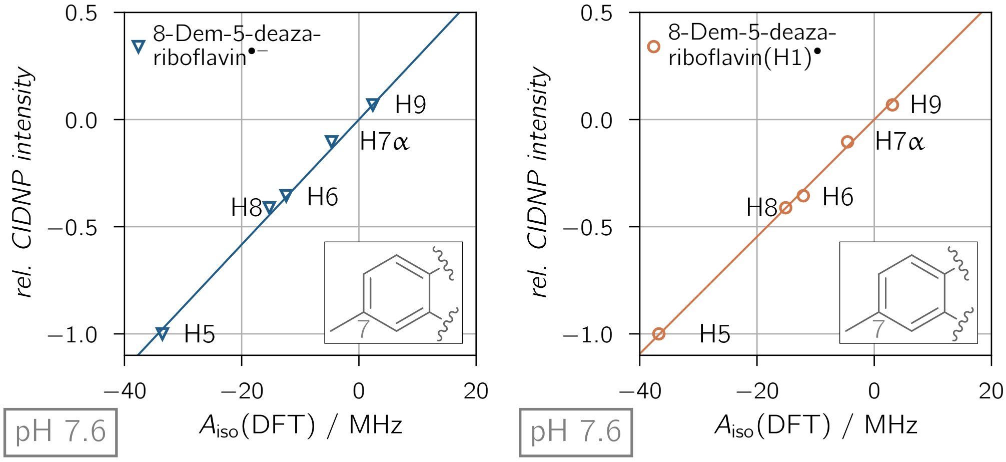

(b) 8-Demethyl-5-deazariboflavin(H1)•: $m = 0.0273\,\mathrm{MHz}^{-1}$, $R^2 = 0.9980$;
8-Demethyl-5-deazariboflavin•−: $m = 0.0292\,\mathrm{MHz}^{-1}$, $R^2 = 0.9959$.

Figure S8 Correlation plots of ^{1}H transient CIDNP experiments of 8-demethyl-5-deazriboflavin (8-dem-5-deazariboflavin) in D_2O at a) pH 1.5 and b) pH 7.6. Linear fits were forced to go through the origin. Values for relative CIDNP intensities were extracted from Figure S4 c) and Figure S5 c) by fitting a Voigt line shape to the CIDNP signals. A_{iso} from DFT calculations are listed in Table S4.

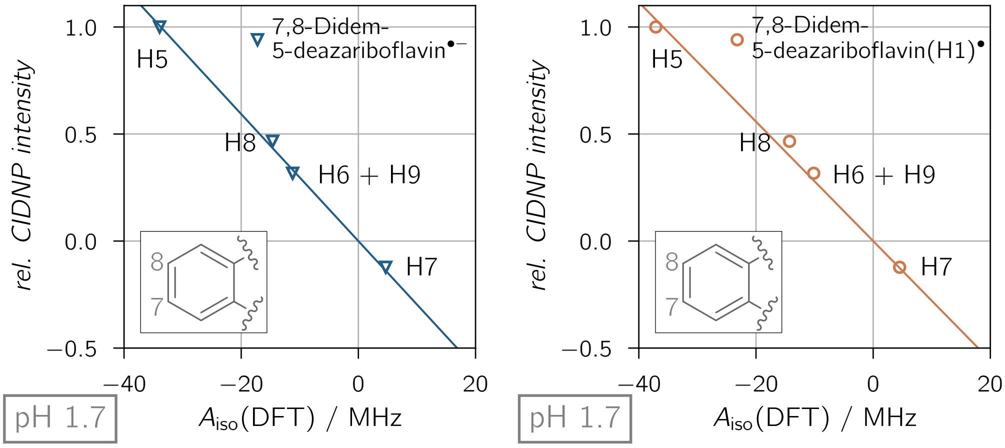

(a) 7,8-Didemethyl-5-deazariboflavin(H1)$^\bullet$: $m = -0.0279\,\text{MHz}^{-1}$, $R^2 = 0.9892$; 7,8-Didemethyl-5-deazariboflavin$^{\bullet-}$: $m = -0.0297\,\text{MHz}^{-1}$, $R^2 = 0.9976$.

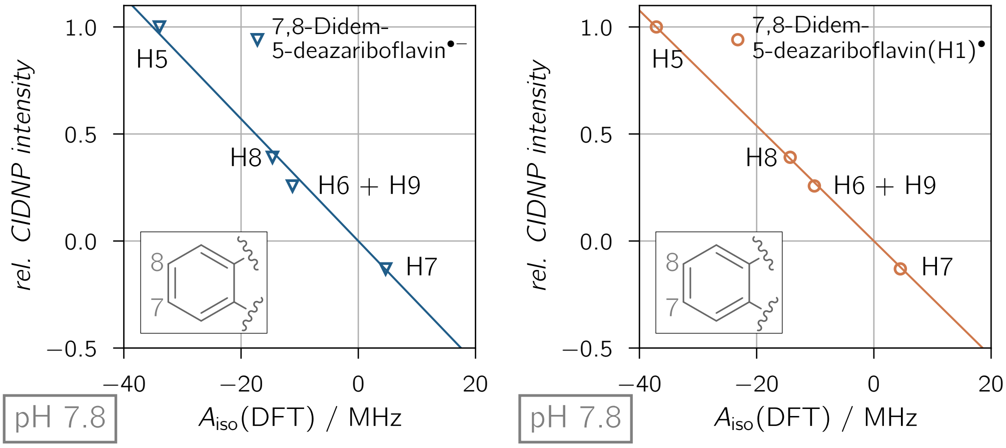

(b) 7,8-Didemethyl-5-deazariboflavin(H1)$^\bullet$: $m = -0.0269\,\text{MHz}^{-1}$, $R^2 = 0.9994$; 7,8-Didemethyl-5-deazariboflavin$^{\bullet-}$:$m = -0.0285\,\text{MHz}^{-1}$, $R^2 = 0.9913$.

Figure S9 Correlation plots of ^{1}H transient CIDNP experiments of 7,8-didemethyl-5-deazariboflavin (7,8-didem-5-deazariboflavin) in D_2O at a) pH 1.7 and b) pH 7.8. Linear fits were forced to go through the origin. Values for relative CIDNP intensities were extracted from Figure S4 d) and Figure S5 d) by fitting a Voigt line shape to the CIDNP signals. The resonances of H6 and H9 are superimposed which is why their CIDNP intensity is given as a sum. A_{iso} from DFT calculations are listed in Table S4.

6 Mulliken Spin Densities of Demethylated 5-Deazariboflavin and Riboflavin Derivatives

Table S5 Mulliken spin densities of neutral and anionic 5-deazariboflavin radicals and demethylated derivatives. All calculations were carried out using an conductor-like continuum model to simulate water solvation. Values are given for nuclei of the 5-deazaisoalloxazine moiety. The neutral and anionic radical species are referred to as Flavin(H1)$^{\bullet}$ and Flavin$^{\bullet-}$, respectively. "Demethyl" is abbreviated by "dem".

	5-Deazariboflavin		7-Dem-5-deazariboflavin		8-Dem-5-deazariboflavin		7,8-Didem-5-deazariboflavin	
Nucleus	Flavin(H1)$^{\bullet}$	Flavin$^{\bullet-}$	Flavin(H1)$^{\bullet}$	Flavin$^{\bullet-}$	Flavin(H1)$^{\bullet}$	Flavin$^{\bullet-}$	Flavin(H1)$^{\bullet}$	Flavin$^{\bullet-}$
C2	0.0223	0.0224	0.0228	0.0269	0.0209	0.0243	0.0214	0.0221
C4	0.0505	0.0626	0.0482	0.0662	0.0494	0.0664	0.0480	0.0601
C4a	−0.0223	0.0129	−0.0263	0.0105	−0.0155	0.0209	−0.0185	0.0183
C5	0.5253	0.4894	0.5267	0.4906	0.5149	0.4763	0.5201	0.4811
C5a	−0.0830	−0.0808	−0.0857	−0.0838	−0.0860	−0.0819	−0.0871	−0.0822
C6	0.1541	0.1661	0.1730	0.1852	0.1604	0.1667	0.1729	0.1814
C7	−0.0670	−0.0708	−0.0725	−0.0756	−0.0729	−0.0743	−0.0772	−0.0800
C7α	0.0045	0.0049	–	–	0.0047	0.0048	–	–
C8	0.1919	0.1995	0.1851	0.1882	0.2091	0.2118	0.1963	0.2016
C8α	−0.0144	−0.0153	−0.0139	−0.0144	–	–	–	–
C9	−0.0672	−0.0598	−0.0612	−0.0508	−0.0609	−0.0498	−0.0562	−0.0465
C9a	0.1247	0.1140	0.1103	0.0975	0.1269	0.1159	0.1169	0.1074
C10a	0.0648	0.0362	0.0734	0.0365	0.0606	0.0279	0.0674	0.0373
H1	0.0002	–	0.0001	–	0.0001	–	0.0001	
H3	0.0002	−0.0001	0.0002	0.0000	0.0002	−0.0001	0.0002	−0.0001
H5	−0.0295	−0.0279	−0.0296	−0.0281	−0.0290	−0.0273	−0.0293	−0.0276
H6	−0.0086	−0.0094	−0.0095	−0.0102	−0.0089	−0.0093	−0.0093	−0.0098
H7	–	–	0.0033	0.0035	–	–	0.0036	0.0038
H7α	−0.0018	−0.0019	–	–	−0.0018	−0.0019	–	–
H8	–	–	–	–	−0.0127	−0.0131	−0.0116	−0.0121

Table S5 Mulliken spin densities of neutral and anionic 5-deazariboflavin radicals and demethylated derivatives. All calculations were carried out using an conductor-like continuum model to simulate water solvation. Values are given for nuclei of the 5-deazaisoalloxazine moiety. The neutral and anionic radical species are referred to as Flavin(H1)$^{\bullet}$ and Flavin$^{\bullet-}$, respectively. "Demethyl" is abbreviated by "dem".

	5-Deazariboflavin		7-Dem-5-deazariboflavin		8-Dem-5-deazariboflavin		7,8-Didem-5-deazariboflavin	
Nucleus	Flavin(H1)$^{\bullet}$	Flavin$^{\bullet-}$	Flavin(H1)$^{\bullet}$	Flavin$^{\bullet-}$	Flavin(H1)$^{\bullet}$	Flavin$^{\bullet-}$	Flavin(H1)$^{\bullet}$	Flavin$^{\bullet-}$
H8α	0.0056	0.0057	0.0058	0.0061	–	–	–	–
H9	0.0029	0.0026	0.0026	0.0022	0.0025	0.0020	0.0023	0.0018
N1	−0.0108	−0.0136	−0.0101	−0.0151	−0.0103	−0.0152	−0.0100	−0.0127
N10	0.0952	0.0850	0.0927	0.0804	0.0962	0.0866	0.0953	0.0845
N3	−0.0116	−0.0073	−0.0122	−0.0097	−0.0109	−0.0078	−0.0114	−0.0073

Table S6 Mulliken spin densities of neutral and anionic riboflavin radicals and demethylated derivatives. All calculations were carried out using an conductor-like continuum model to simulate water solvation. Values are given for nuclei of the isoalloxazine moiety. The neutral and anionic radical species are referred to as Flavin(H1)$^{\bullet}$ and Flavin$^{\bullet-}$, respectively. "Demethyl" is abbreviated by "dem".

	Riboflavin		7-Dem-riboflavin		8-Dem-riboflavin		7,8-Didem-riboflavin	
Nucleus	Flavin(H1)$^{\bullet}$	Flavin$^{\bullet-}$	Flavin(H1)$^{\bullet}$	Flavin$^{\bullet-}$	Flavin(H1)$^{\bullet}$	Flavin$^{\bullet-}$	Flavin(H1)$^{\bullet}$	Flavin$^{\bullet-}$
C2	0.0045	0.0186	0.0048	0.0191	0.0040	0.0178	0.0043	0.0186
C4	0.0234	0.0521	0.0232	0.0514	0.0219	0.0507	0.0216	0.0501
C4a	0.2368	0.0884	0.2413	0.0889	0.2513	0.0983	0.2573	0.0988
C5a	−0.0052	0.0568	−0.0064	−0.0589	−0.0143	−0.0626	−0.0161	−0.0635
C6	0.0498	0.1177	0.0579	0.1259	0.0533	0.1225	0.0641	0.1346
C7	−0.0054	−0.0473	−0.0071	−0.0508	−0.0130	−0.0530	−0.0153	−0.0561

Table S6 Mulliken spin densities of neutral and anionic riboflavin radicals and demethylated derivatives. All calculations were carried out using an conductor-like continuum model to simulate water solvation. Values are given for nuclei of the isoalloxazine moiety. The neutral and anionic radical species are referred to as Flavin(H1)$^{\bullet}$ and Flavin$^{\bullet-}$, respectively. "Demethyl" is abbreviated by "dem".

	Riboflavin		7-Dem-riboflavin		8-Dem-riboflavin		7,8-Didem-riboflavin	
Nucleus	Flavin(H1)$^{\bullet}$	Flavin$^{\bullet-}$	Flavin(H1)$^{\bullet}$	Flavin$^{\bullet-}$	Flavin(H1)$^{\bullet}$	Flavin$^{\bullet-}$	Flavin(H1)$^{\bullet}$	Flavin$^{\bullet-}$
C7α	0.0004	0.0031	–	–	0.0011	0.0035	–	–
C8	0.1051	0.1545	0.0959	0.1477	0.1085	0.1660	0.0971	0.1546
C8αa	−0.0071	−0.0115	−0.0061	−0.0106	–	–	–	–
C9	−0.0435	−0.0559	−0.0403	−0.0531	−0.0372	−0.0492	−0.0336	−0.0454
C9a	0.0871	0.1131	0.0789	0.1072	0.0879	0.1142	0.0769	0.1036
C10a	−0.0063	0.0357	−0.0038	0.0382	−0.0077	0.0332	−0.0049	0.0369
H3	−0.0009	−0.0003	−0.0009	−0.0003	−0.0010	−0.0003	−0.0009	−0.0003
H5	−0.0139	–	−0.0139	–	−0.0136	–	−0.0136	–
H6	−0.0031	−0.0068	−0.0035	−0.0070	−0.0032	−0.0069	−0.0037	−0.0073
H7	–	–	−0.0001	0.0022	–	–	0.0005	0.0026
H7α	0.0000	−0.0013	–	–	−0.0003	−0.0014	–	–
H8	–	–	–	–	−0.0063	−0.0100	−0.0055	−0.0091
H8α	0.0033	0.0047	0.0029	0.0043	–	–	–	–
H9	0.0016	0.0024	0.0015	0.0022	0.0012	0.0019	0.0011	0.0018
N1	0.0020	−0.0090	0.0046	−0.0081	0.0029	−0.0085	0.0064	−0.0072
N10	0.1464	0.1187	0.1424	0.1172	0.1488	0.1207	0.1426	0.1174
N3	0.0080	−0.0042	0.0073	−0.0046	0.0086	−0.0035	0.0078	−0.0042
N5	0.2994	0.3813	0.3011	0.3832	0.2930	0.3721	0.2950	0.3748

References

(1) Pompe, N.; Chen, J.; Illarionov, B.; Panter, S.; Fischer, M.; Bacher, A.; Weber, S. *J. Chem. Phys.* **2019**, *151*.

(2) van Schie, M. M. C. H.; Younes, S. H. H.; Rauch, M. C. R.; Pesic, M.; Paul, C. E.; Arends, I. W. C. E.; Hollmann, F. *Mol. Catal.* **2018**, *452*, 277–283.
